# A Giant Pliosaurid Skull from the Late Jurassic of England

**DOI:** 10.1371/journal.pone.0065989

**Published:** 2013-05-31

**Authors:** Roger B. J. Benson, Mark Evans, Adam S. Smith, Judyth Sassoon, Scott Moore-Faye, Hilary F. Ketchum, Richard Forrest

**Affiliations:** 1 Department of Earth Sciences, University of Oxford, Oxford, United Kingdom; 2 New Walk Museum and Art Gallery, Leicester Arts and Museums Service, Leicester, United Kingdom; 3 Nottingham Natural History Museum, Nottingham, United Kingdom; 4 School of Earth Sciences, University of Bristol, Bristol, United Kingdom; 5 Wavecut Platform Ltd., Sevenoaks, United Kingdom; 6 University Museum of Zoology, University of Cambridge, Cambridge, United Kingdom; 7 Sedgwick Museum of Earth Sciences, University of Cambridge, Cambridge, United Kingdom; 8 Radcliffe-on-Trent, Nottinghamshire, United Kingdom; Ludwig-Maximilians-Universität München, Germany

## Abstract

Pliosaurids were a long-lived and cosmopolitan group of marine predators that spanned 110 million years and occupied the upper tiers of marine ecosystems from the Middle Jurassic until the early Late Cretaceous. A well-preserved giant pliosaurid skull from the Late Jurassic Kimmeridge Clay Formation of Dorset, United Kingdom, represents a new species, *Pliosaurus kevani*. This specimen is described in detail, and the taxonomy and systematics of Late Jurassic pliosaurids is revised. We name two additional new species, *Pliosaurus carpenteri* and *Pliosaurus westburyensis*, based on previously described relatively complete, well-preserved remains. Most or all Late Jurassic pliosaurids represent a globally distributed monophyletic group (the genus *Pliosaurus*, excluding ‘*Pliosaurus*’ *andrewsi*). Despite its high species diversity, and geographically widespread, temporally extensive occurrence, *Pliosaurus* shows relatively less morphological and ecological variation than is seen in earlier, multi-genus pliosaurid assemblages such as that of the Middle Jurassic Oxford Clay Formation. It also shows less ecological variation than the pliosaurid-like Cretaceous clade Polycotylidae. Species of *Pliosaurus* had robust skulls, large body sizes (with skull lengths of 1.7–2.1 metres), and trihedral or subtrihedral teeth suggesting macropredaceous habits. Our data support a trend of decreasing length of the mandibular symphysis through Late Jurassic time, as previously suggested. This may be correlated with increasing adaptation to feeding on large prey. Maximum body size of pliosaurids increased from their first appearance in the Early Jurassic until the Early Cretaceous (skull lengths up to 2360 mm). However, some reduction occurred before their final extinction in the early Late Cretaceous (skull lengths up to 1750 mm).

## Introduction

Pliosaurids were Mesozoic ocean predators, some of which achieved giant sizes >12 metres long [1]–[3]. They had a global distribution and spanned c. 110 million years, from the Early Jurassic until the early Late Cretaceous (e.g., [4]–[14]). However, their fossils are best known from the Late Jurassic Oxford and Kimmeridge Clay formations of England (e.g., [15]–[17]). Pliosaurids form part of a larger clade of marine reptiles, Plesiosauria, characterised by highly plastic body proportions, and including extremely long-necked taxa such as *Elasmosaurus*, as well as short-necked, large-skulled taxa informally termed ‘pliosauromorphs’ [18]–[19].

Early pliosaurid evolution shows a transition from plesiomorphic, intermediate body proportions and a small skull in the earliest Early Jurassic taxa [13], [19] to Middle Jurassic taxa with ‘pliosauromorph’ body proportions and piscivorous (gracile, longirostine skulls, many slender teeth) or macropredaceous habits (robust longirostrine or brevirostrine skulls with robust teeth) (e.g., [20]–[25]). These Middle Jurassic and younger taxa belong to an easily recognisable clade called Thalassophonea [26]. Pliosaurid diversity declined in the Late Jurassic, leaving only macropredaceous forms [26]. The latest pliosaurids are from the early Late Cretaceous [14], [27], and they may have been made extinct by the appearance of large-bodied mosasauroids as competitors in the Middle Turonian [14].

The first pliosaurid fossil discoveries were from the English, Late Jurassic Kimmeridge Clay Formation. In 1822, Conybeare figured vertebrae from near Weymouth and mentioned similar remains from Headington Pits, near Oxford ([28]:plate 22, figs 4–8). Later, in 1824, he also mentioned the fragmentary remains of a large-bodied, short necked plesiosaurian in William Buckland's collection, from Market Rasen in Lincolnshire ([29]:p. 389 “Market Raisin”) and provisionally suggested the name *Plesiosaurus giganteus* (a *nomen oblitum*) for short-necked plesiosaurians in general. In 1841, Owen erected the subgenus ‘*Pleiosaurus*’ for a new species *Plesiosaurus* (*Pleiosaurus*) *brachydeirus* [30]. Owen mentioned two specimens, the Market Rasen skeleton (OXFUM (Oxford University Museum of Natural History, Oxford, United Kingdom) J.9245, J.9247–J.9301, J.10453, comprising a partial skull and postcrania) and a tooth from the Kimmeridge Clay Formation of Shotover, near Oxford ([30]: p. 282–285, plate 68, figs 5–5″). One year later, in 1842, Owen emended the subgenus ‘*Pleiosaurus*’ to the genus *Pliosaurus* ([31]:p. 60–64), which has been followed by all subsequent authors (e.g., [15], [17], [32]–[34]) except Phillips in 1871 [35]. Owen [31] stated that *Pliosaurus brachydeirus* was intended as the name for the Market Rasen specimen (OXFUM J.9245 etc.), and described additional fragmentary remains and isolated bones and teeth from the Kimmeridge Clay Formation at various British localities.

Since these early reports, in addition to further discoveries from the United Kingdom, Late Jurassic pliosaurid remains have been collected from France [36], North America [4], [37], Russia [7]–[9], [38]–[40], Mexico [2], Cuba [12] and Svalbard [3], including several valid species. In spite of the global distribution of these finds, they document an ecologically and taxonomically conservative assemblage of large-bodied (adult specimens suggest body lengths estimated from 10–12 metres; e.g., [2]–[3]), robust skulled, long-snouted pliosaurids with distinctive, trihedral teeth [17], [26], [34] (a gracile, longirostrine ecotype from Italy may be early Late Jurassic or late Middle Jurassic in age [41]). Although various taxonomists have recognised multiple Late Jurassic pliosaurid genera (e.g., [9], [12], [17], [40], [42]–[43]), in this paper we suggest that most, perhaps all, specimens form a monophyletic group representing a single genus, *Pliosaurus*. This was also suggested for Kimmeridgian–Tithonian taxa by Knutsen [34], and contrasts sharply with the high genus-level and ecological diversity of pliosaurids seen in the late Middle Jurassic (Callovian) Peterborough Member of the Oxford Clay Formation [16], [23]–[24], [26], [44]. The morphologically and ecologically restricted nature of Late Jurassic (and younger [26]) pliosaurids also contrasts with the variety of the pliosaurid-like Cretaceous clade Polycotylidae. Polycotylids are especially abundant in the Late Cretaceous and include primarily longirostrine forms with gracile snouts and either slender, widely spaced, isodont teeth indicative of piscivory, or a slightly more robust snout and dentition suggesting intermediate levels of macropredation [45]–[49].

Since the early pliosaurid discoveries described above, numerous additional pliosaurid remains have been reported from the Kimmeridge Clay Formation. Many are isolated bones and teeth or skeletal fragments (e.g., [31], [43], [50]–[51]). However, others comprise more complete cranial remains, some with associated postcrania [15], [42], [52]–[55], and partial postcranial skeletons [56]–[57]. Although many species of Kimmeridge Clay Formation pliosaurid have been erected, only three have recently been considered valid [17], [34], [54]: *Pliosaurus brachydeirus*, *P*. *brachyspondylus* and *P. macromerus*. Of these, the name-bearing type specimens of *P. brachyspondylus* (Owen, 1840) [58] (a neotype) and *P. macromerus* Phillips, 1871 [35] (a lectotype) are single vertebrae [17], [52], [57]. These vertebrae were considered undiagnostic in the most recent taxonomic appraisal of *Pliosaurus* [34]. In consequence, even the more complete and informative pliosaurid skulls and skeletons are in taxonomic limbo [34], [42], [51], [54]–[55]. Thus, substantial work remains to understand pliosaurid diversity and evolution in the Kimmeridge Clay Formation.

**Figure 1:**
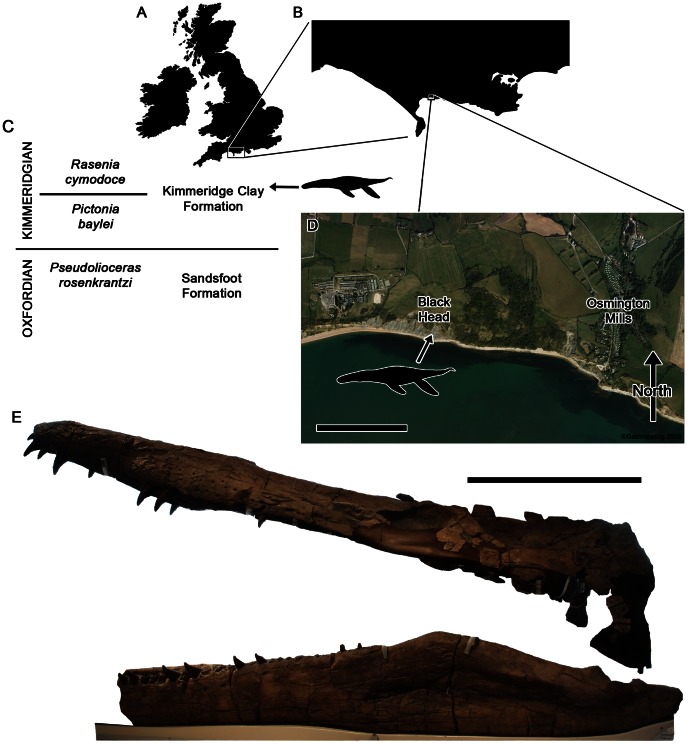
Locality and horizon of *Pliosaurus kevani* n. sp. DORCM G.13,675. Maps of the United Kingdom (A), Dorset area (B), and Osmington Bay (D) showing the locality of the specimen, indicated by a pliosaurid silhouette. The stratigraphic context of the find (C), the approximate level of the Wyke Siltstone bed is marked by a pliosaurid silhouette and the stratigraphic section at Black Head was given in full by ([63]:fig. 6). The mounted skull of DORCM G.13,675 in left lateral view (E). Scale bars equal 500 m (D) and 500 mm (E).

In this paper we report DORCM (Dorset County Museum, Dorchester, United Kingdom) G.13,675, a new, near-complete, pliosaurid skull from the Kimmeridge Clay Formation of Osmington Bay, Dorset, United Kingdom (the locality is shown in Fig. 1). Distinctive anatomical features suggest that DORCM G.13,675 represents a new species of *Pliosaurus*, which we name *Pliosaurus kevani* n. sp. (see *Systematic Palaeontology*). The discovery of *P. kevani* presents an opportunity to clarify the cranial anatomy and taxonomy of *Pliosaurus*. We present a complete description of this specimen, and taxonomic reappraisal of the genus in which we provide differential diagnoses and species names for the most complete specimens in order to stabilize the taxonomy of *Pliosaurus*.

## Methods

### Collection

DORCM G.13,675 was collected over a period of eight years as pieces up to 60 kg in mass weathered out of the sea-cliff. Most were collected by Kevan Sheehan, the owner of a small café overlooking the sea at Osmington Mills during daily walks along the foreshore. No permits were required for the collection of most of these pieces, which occurred in loose, fallen blocks in the intertidal zone. Other parts were collected in situ from privately owned land, and were purchased from the land owner. They were first identified as a pliosaurid skull by Richard Edmonds, Earth Sciences Manager for Dorset and East Devon Coast World Heritage Site Team. Steve Etches, a well-known local collector of Kimmeridge Clay Formation fossils identified the stratigraphic horizon from which the specimen was eroding and assisted in recovering some posterior parts of the skull. The specimen was then purchased with funding secured by David Tucker of Dorset County Council's museum service from the Heritage Lottery Fund Collecting Cultures programme and Dorset and Devon county councils.

The impressive size and completeness of DORCM G.13,675 has generated significant media coverage. Its acquisition was announced publicly in October 2009. Some additional pieces came to light. These were donated by Patrick Clarke and purchased from Shirley Swaine. DORCM G.13,675 was prepared and mounted by S. M.-F. (see *Specimen Preparation*), and went on display in Dorchester County Museum in July 2011 with an official opening by Sir David Attenborough.

### Specimen preparation

Preparation of DORCM G.13,675 was undertaken between March 2010 and March 2011. The bulk of the matrix was removed using a modified Chicago pneumatic air pen. On areas where the matrix was particularly thick or where it was obvious that the underlying bone lay as a continuous sheet, a series of thin 5 mm deep slots were cut at 90 degrees to each other with a 40 mm diamond blade and the resulting blocks were chipped off using either an airpen or chisel. An airbrasive unit equipped with 50 micron aluminium oxide abrasive powder was used to remove the remaining matrix from the surface of the bone. The broken surface produced by the airpen made it difficult to identify the encrusting epifauna, so a 100 mm angle grinder fitted with a continuous diamond blade was used on these areas to grind away the matrix instead. The encrusting oysters appeared as a series of white circles within the grey matirx. This method proved very effective, because once the encrusting epifauna had been identified and delimited it was possible to finish the preparation of these areas with the airpen and airbrasive unit. The average mass of the sections making up the lower jaws was around 20 kg. The mass of the sections comprising the skull ranged from 15 kg upwards. Over 30 kg of matrix was removed from the block containing the orbits, after which this block weighed 50 kg. Load bearing breaks were bonded with the epoxy resin adhesive Araladite 2012. Preparation of the lower jaws took 200 hours and a further 365 hours were needed to complete preparation of the skull.

### Nomenclatural acts

The electronic edition of this article conforms to the requirements of the amended International Code of Zoological Nomenclature, and hence the new names contained herein are available under that Code from the electronic edition of this article. This published work and the nomenclatural acts it contains have been registered in ZooBank, the online registration system for the ICZN. The ZooBank LSIDs (Life Science Identifiers) can be resolved and the associated information viewed through any standard web browser by appending the LSID to the prefix "http://zoobank.org/". The LSID for this publication is: urn:lsid:zoobank.org:pub:D3EE687F-52BF-4111-9203-280BFAA40F63. The electronic edition of this work was published in a journal with an ISSN, and has been archived and is available from the following digital repositories: PubMed Central and LOCKSS.

## Results

### Systematic palaeontology

Sauropterygia Owen, 1860 [59]

Plesiosauria de Blainville, 1835 [60]

Pliosauridae Seeley, 1874 [61]

Thalassophonea Benson & Druckenmiller, 2013 [26]

Genus *Pliosaurus* Owen, 1841 [30]

1824 *Plesiosaurus* Conybeare; Conybeare 1824 [29]:p. 389 (as *Plesiosaurus giganteus*, a *nomen oblitum*)

1841 *Plesiosaurus* (*Pleiosaurus*) Owen; Owen 1841 ([30]:p. 282-285, plate 68, figs 5–5″)

1842 *Pliosaurus* Owen; Owen 1842 ([31]:p. 60–65)

1871 *Pleiosaurus* Owen; Phillips 1871 ([35]:p. 316–318, 341–366)

1959 *Stretosaurus* Tarlo; Tarlo 1959 ([57]:p. 40) (subjective junior synonym)

1989 *Liopleurodon* Sauvage; Halstead 1989 ([42]:p. 38) (as *Liopleurodon macromerus*)

#### Type species

Pliosaurus brachydeirus Owen, 1841 [30]

#### Other included valid species

Pliosaurus kevani n. sp.; Pliosaurus westburyensis n. sp.; Pliosaurus carpenteri n. sp.; Pliosaurus rossicus Novozhilov, 1948a [7]; Pliosaurus funkei Knutsen et al., 2012 [3].

#### Diagnosis

Pliosaurids possessing seven autapomorphies: (1) trihedral or subtrihedral teeth (although similar teeth are also present in *Gallardosaurus iturraldei* from the Oxfordian of Cuba [12], which may or may not be referable to *Pliosaurus*; see Phylogenetic analysis); (2) anterior end of premaxilla–maxilla contact on lateral surface of snout deeply interdigitating with an anteroposteriorly ‘zig-zagging’ appearance; (3) occipital condyle lacking notochordal pit, but scored by several, irregularly-arranged grooves; (4) first (mesialmost) premaxillary alveolus reduced to approximately half or less the diameter of the second alveolus (although an even smaller, perhaps vestigial, first alveolus may be present in some Cretaceous pliosaurids [27]); (5) long posteroventral process of the jugal ventrally underlaps the squamosal; (6) dorsal surface of surangular mediolaterally broad, as in other thalassophonean pliosaurids, but inclined to face dorsolaterally (except in *Pliosaurus carpenteri* n. sp.) and bounded laterally by an anteroposteriorly oriented groove, unlike in other pliosaurids (this groove is absent in *P. carpenteri* and an immature specimen proposed as the ‘neotype’ of *Pliosaurus brachyspondylus* by Knutsen [34], CAMSM (Sedgwick Museum of Earth Sciences, Cambridge, United Kingdom) J.35991); (7) proximal surfaces of radius and tibia markedly convex in large individuals (possibly controlled by ontogeny and absent in immature specimens such as CAMSM J.35991 and the holotype of *Pliosaurus brachydeirus*).

#### Remarks

Several authors have noted that *Plesiosaurus* (*Pleiosaurus*) Owen, 1841 [30] (a subgenus) is the original spelling of *Pliosaurus* (a genus) (e.g., [34]). Although ‘*Pleiosaurus*’ is the correct original spelling (in the sense of Article 32 of the ICZN), as far as we know, no authors have used this spelling since Phillips in 1871 [35]. Instead, ‘*Pliosaurus*’, is in prevailing usage (e.g., [17], [34], [42]–[43], [54]–[55]), and should be preserved (Article 33.3.1 of the ICZN).

The taxonomy of *Pliosaurus* was reviewed by Tarlo in 1960 [17] and Knutsen in 2012 [34], both establishing that several historic taxa are based on undiagnostic type materials and are *nomina dubia*. We do not repeat all such details here, and concur with many of the statements of Knutsen [34]. For example, observation of taxonomically important anatomical variation among *Pliosaurus* specimens with intermediate counts of mandibular symphysial alveoli (8–10) causes us to agree that *Pliosaurus portentificus* Noè et al., 2004 [43] is a *nomen dubium* referrable to *Pliosaurus* indet [34]. We also agree that diagnostic features of *Pliosaurus irgisensis* (Novozhilov, 1948) [7] have yet to be established and that its type specimen requires redescription. This taxon should be considered a *nomen dubium*, and its type specimen referred to Thalassophonea indet. However, several differences do exist between our assessment of *Pliosaurus* taxonomy and that of Knutsen [34]. These differences are explained here and in the sections below.

The type specimens of several nominal taxa are based on specifically undiagnostic remains and represent *nomina dubia*. For example *Pliosaurus brachyspondylus* (Owen, 1840) [58], based on a neotypic cervical vertebra (CAMSM J.29564 [52]), and *Pliosaurus macromerus* (Phillips, 1871) [35], based on a lectotypic cervical vertebra (OXFUM J.10441 [57]). Knutsen [34] proposed replacement type specimens for these species, pending a petition to the ICZN that has not yet been made (E. M. Knutsen, pers. comm. March 2013). Although we consider the proposed replacement types of these historical taxa to be diagnostic and distinct from the species proposed in the current work, until the appeal is made, *P. brachyspondylus* and *P. macromerus* are *nomina dubia*. Their current name-bearing type specimens should be considered Thalassophonea indet.

Knutsen [34] suggested possible synonymy between *Pliosaurus macromerus* and *P. rossicus* based on the presence of only six symphysial and five premaxillary alveoli in both [7], [17], [34], [40]. He also proposed NHMUK (Natural History Museum, London, United Kingdom) PV OR 39362 as the replacement type of *P. macromerus*, pending a petition to the ICZN [34]. NHMUK PV OR 39362 is a partial skull, first described in 1869 [15]. However, although NHMUK PV OR 39362 was said by Knutsen [34] and earlier authors [17], [43], [57] to have approximately six symphysial alveoli, in fact seven are present as preserved, and some mesial alveoli are missing. Furthermore, we estimate that a total of nine were present prior to breakage (pers. obs., NHMUK PV OR 39362). Although another specimen referred to *P. macromerus*, OXFUM J.10454, does have a short symphysis containing only six alveoli ([17]:plate 22, fig. 5), the marked difference in symphysial tooth counts indicates that OXFUM J.10454 is distinct from NHMUK PV OR 39362. Thus, we consider *P. rossicus* to be a valid species of *Pliosaurus*, based on the presence of a short symphysis containing only six alveoli, and provisionally refer OXFUM J.10454 to *P.* ?*rossicus* on the basis of this feature.

#### Specimens referred to *Pliosaurus* indet

In addition to the holotype of *Pliosaurus portentificus* (discussed above and in [34]), several other specimens can be referred to *Pliosaurus* but are not diagnostic at the species level. Many of these are isolated trihedral teeth (e.g., [30], [50]). Isolated cervical vertebrae (e.g., [28]–[29], [31]) are not diagnostic except to Pliosauridae indet. We do not discuss all fragmentary material in detail here, but focus on key specimens.

BHN (Musée-sur-Mer, Boulogne, France) 2R.370, a mandible from the Kimmeridgian of Moulin-Wibert quarry, Boulonnais, France [36], [62] is referred to *Pliosaurus* based on possession of a broad, dorsolaterally facing surangular fossa, bounded laterally by a fossa and ridge. This specimen was originally referred to *Pliosaurus grandis* [62] and later to *P. brachyspondylus* by [36], based on its count of 9–10 symphysial alveoli. However, *Pliosaurus brachyspondylus* is currently a *nomen dubium*, and it is not clear that when its taxonomy is clarified [34], that intermediate symphysial alveolar counts (of 8–10 alveoli) will be useful in species determination within *Pliosaurus*. We note that *Pliosaurus carpenteri* n. sp. has a similar count of symphysial alveoli (nine) to that proposed for *P. brachyspondylus* [17], [34], [43], but differs from CAMSM J.35991, the proposed replacement type of *P. brachyspondylus* [34], and from BHN 2R.370, in possessing an autapomorphic morphology of the surangular (see the *Diagnosis* of *P. carpenteri*). Thus, BHN 2R.370 cannot be identified to species level based on currently available information and should be considered *Pliosaurus* indet. A similar situation pertains to SEKC.K1.2 (Steve Etches Kimmeridge collection ( =  Museum of Jurassic Marine Life)), a mandible with eight symphysial alveoli referred to *Liopleurodon macromerus* by Clarke & Etches [53] and *Pliosaurus portentificus* by Noè et al. [43]. SEKC.K1.2 should be considered as *Pliosaurus* indet. unless other information on its morphology becomes available.

### Pliosaurus brachydeirus Owen, 1841 [30]

1841 *Plesiosaurus* (*Pleiosaurus*) *brachydeirus* Owen; Owen 1841 ([30]:pp. 282–285, pl. 68, figs 5–5″)

1842 *Pliosaurus brachydeirus* Owen; Owen 1842 ([31]:p. 64)

1871 *Pleiosaurus brachydeirus* Owen; Phillips 1871 ([35]:pp. 341–354, figs 134–135)

#### Holotype and only specimen

OXFUM, J.9245, J.9247–J.9301, J.10453, a partial skull and postcranial skeleton.

#### Locality and horizon

*Rasenia cymodoce* Biozone [34] (Lower Kimmeridgian) of the Kimmeridge Clay Formation of Market Rasen, Lincolnshire, United Kingdom.

#### Diagnosis

Species of *Pliosaurus* with the following unique character combination: high dentary alveolar count including 24 postsymphysial alveoli (>35 total) and an estimated total count of 36–37; high count of symphysial dentary alveoli (>11), estimated as 12–13; fully trihedral teeth; mediolateral expansion of premaxilla and maxillary caniniform region relatively slight; six closely-spaced premaxillary alveoli; distalmost premaxillary alveolus similar in size to more mesial alveoli (i.e. non-‘anisodont’ or non-‘heterodont’ premaxillary dentition); diastema present between maxillary and premaxillary alveolar rows; premaxilla–parietal suture located level with the anterior region of the orbit; broad, low, anteroposteriorly oriented ridge on ventral surfaces of cervical centra; epipodials with flat proximal articular surfaces (although this may result from the subadult ontogenetic status of the holotype and only specimen).

### *Pliosaurus kevani* n. sp

urn:lsid:zoobank.org:act:39B2679D-B3FD-4DBA-B5F1-196294DB03D0

#### Holotype and only specimen

DORCM G.13,675, a skull (Figs 1–22)

**Figure 2:**
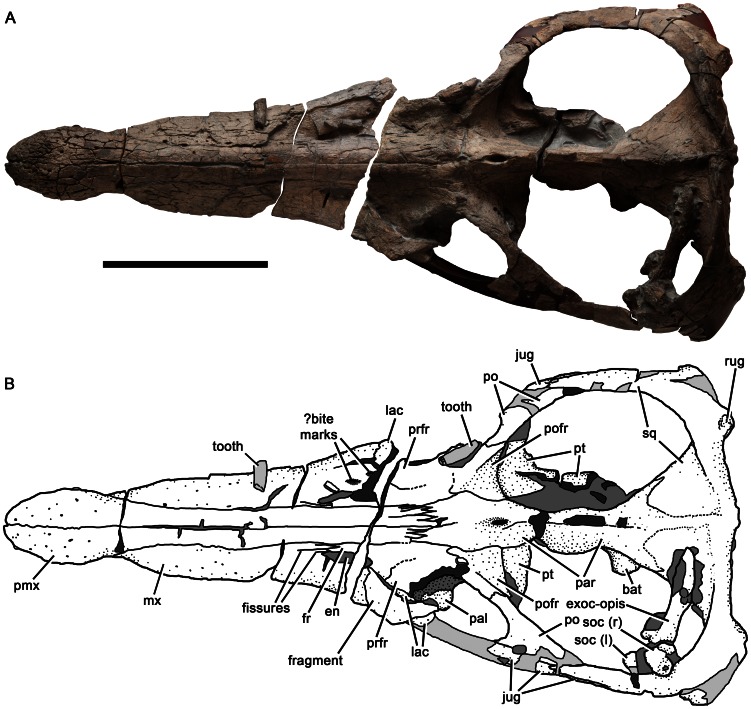
Cranium of *Pliosaurus kevani* n. sp. DORCM G.13,675 in dorsal view (A–B). In line drawing (B) dark grey tone represents broken bone surface, mid grey represents matrix, and light grey represents tooth or artificial restoration. Abbreviations: bat, basal tuber; en, external naris; exoc-opis, exoccipital-opisthotic; fr, frontal; jug, jugal; lac, ‘lacrimal’; mx, maxilla; pal, palatine; par parietal; pmx, premaxilla; po, postorbital; pofr, postfrontal; prfr, prefrontal; pt, pterygoid; rug, rugose eminence; soc (l), left portion of supraoccipital; soc (r), right portion of supraoccipital; sq, squamosal. Ss

**Figure 3:**
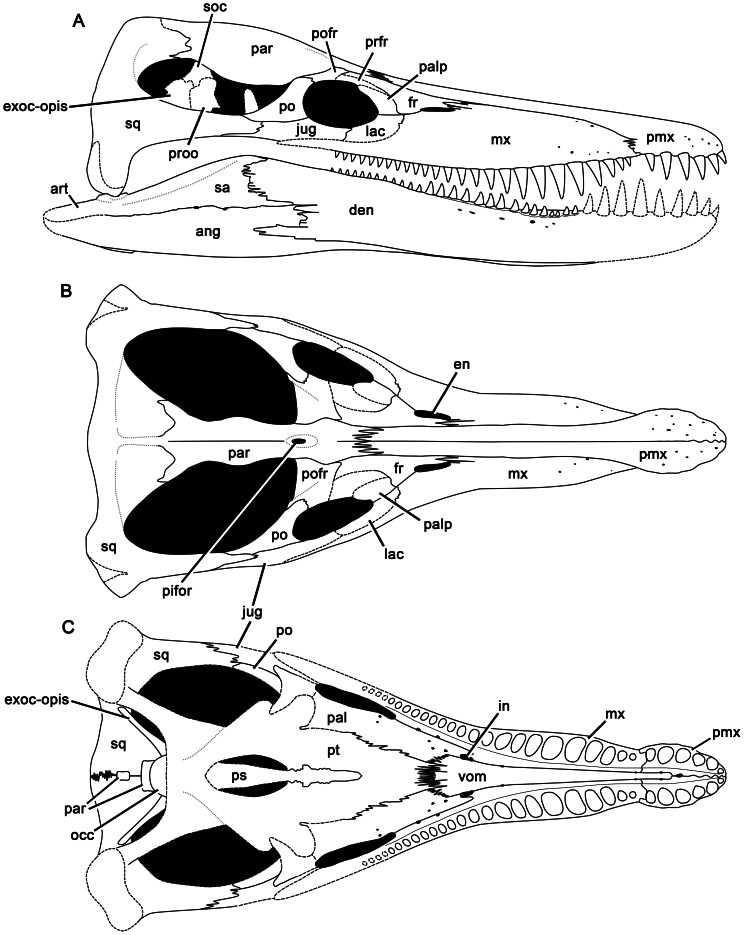
Reconstruction of the skull of *Pliosaurus kevani* n. sp. In right lateral (A), dorsal (B), and ventral (C) views. Abbreviations: ang, angular; art, articular; den, dentary; en, external naris; exoc-opis, exoccipital-opisthotic; in, internal naris; jug, jugal; fr, frontal; lac, ‘lacrimal’; mx, maxilla; occ, occipital condyle; pal, palatine; palp, palpebral; par, parietal; pifor, pineal foramen; pmx, premaxilla; po, postorbital; pofr, postfrontal; prfr, prefrontal; proo, prootic; ps, parasphenoid; pt, pterygoid; sa, surangular; soc, supraoccipital; sq, squamosal; vom, vomer. Total skull length is approximately 2 metres.

**Figure 4:**
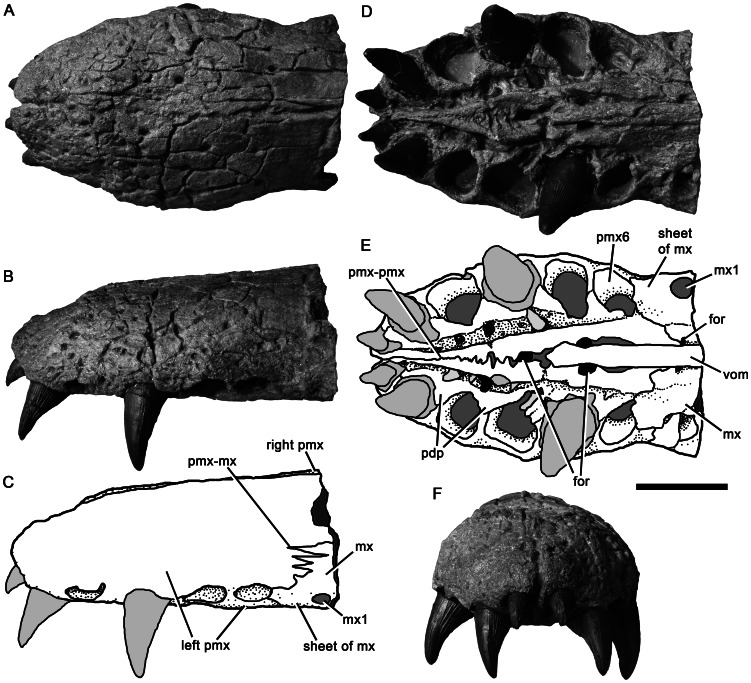
Premaxilla of *Pliosaurus kevani* n. sp. DORCM G.13,675 and contacting portions of the maxilla. In dorsal (A), left lateral (B–C), ventral (D–E), and anterior (F) views. In line drawings (C, E) dark grey tone represents broken bone surface, mid grey represents matrix, and light grey represents tooth or artificial restoration. Abbreviations: for, foramen; mx, maxilla; mx1, first maxillary alveolus; pdp, paradental plates; pmx, premaxilla; pmx6, sixth premaxillary alveolus; pmx-mx, premaxilla-maxilla contact; pmx-pmx, premaxilla-premaxilla contact; vom, vomer. Scale bar equals 100 mm

**Figure 5:**
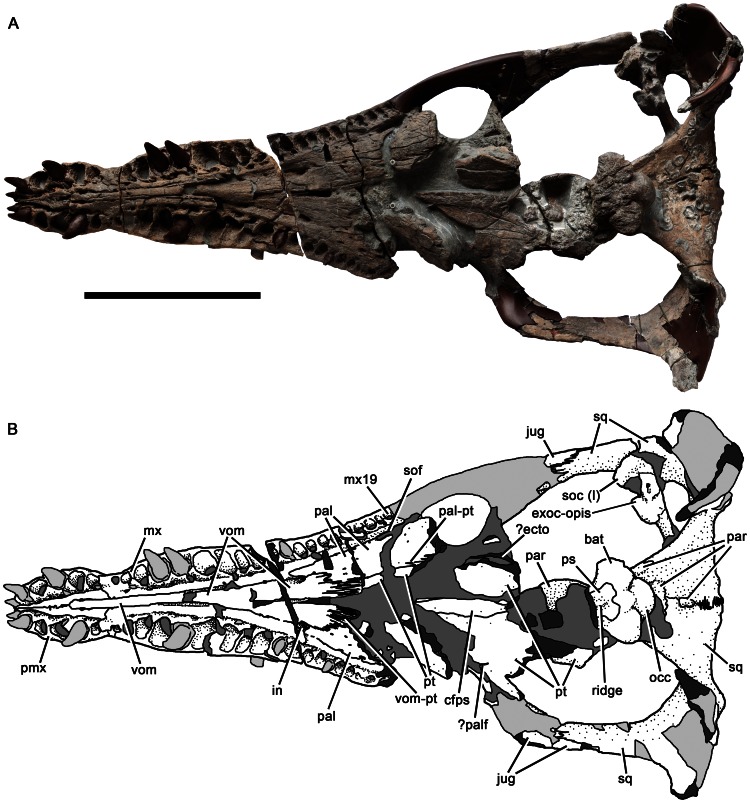
Cranium of *Pliosaurus kevani* n. sp. DORCM G.13,675 in ventral view. In line drawing (B) dark grey tone represents broken bone surface, mid grey represents matrix, and light grey represents tooth or artificial restoration. Abbreviations: bat, basal tuber; cfps, cultriform process of the parasphenoid; ecto, ectopterygoid; exoc-opis, exoccipital-opisthotic; in, internal naris; jug, jugal; mx, maxilla; mx19, 19^th^ maxillary alveolus; occ, occipital condyle; pal, palatine; pal-pt, palatine-pterygoid contact; palf, palatal fenestra; par, parietal; pmx, premaxilla; ps, parasphenoid; pt, pterygoid; soc (l), left portion of supraoccipital; sof, suborbital fenestra; sq, squamosal; vom, vomer; vom-pt, vomer-pterygoid contact. Scale bar equals 500 mm.

**Figure 6:**
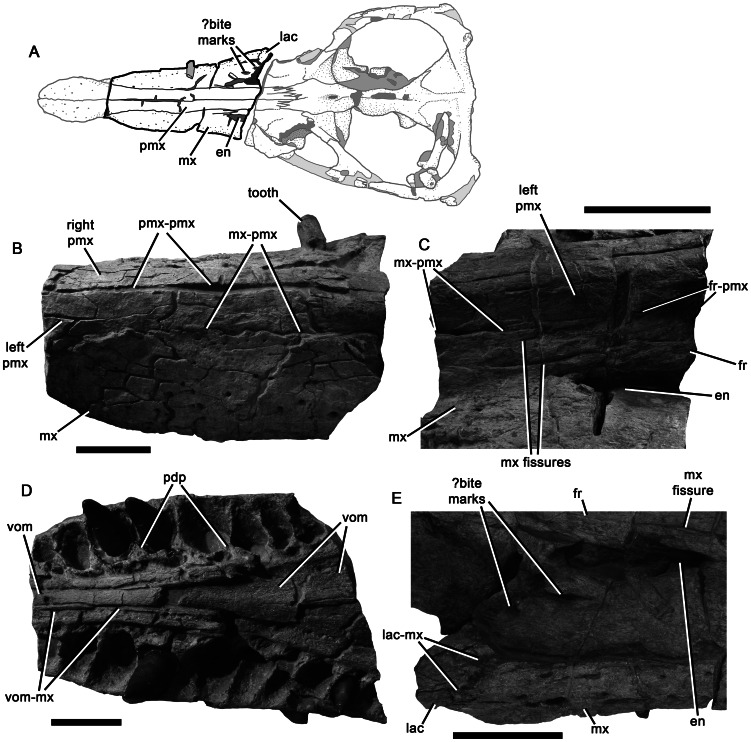
Maxilla of *Pliosaurus kevani* n. sp. DORCM G.13,675 and contacting bones. Schematic (A) showing the anterior and posterior portions of the maxilla figured in (B–E). Anterior portion in left dorsolateral (B) and ventral (D) views, and posterior portion in left dorsolateral (C) and right dorsolateral (E) views. Abbreviations: en, external naris; fr, frontal; fr-pmx, frontal, premaxilla contact; mx, maxilla; mx-pmx, maxilla-premaxilla contact; lac, ‘lacrimal’; lac-mx, ‘lacrimal’-maxilla contact; pdp, paradental plates; pmx, premaxilla; pmx-pmx, premaxilla-premaxilla contact; vom, vomer; vom-mx, vomer-maxilla contact. Scale bars equal 100 mm.

**Figure 7:**
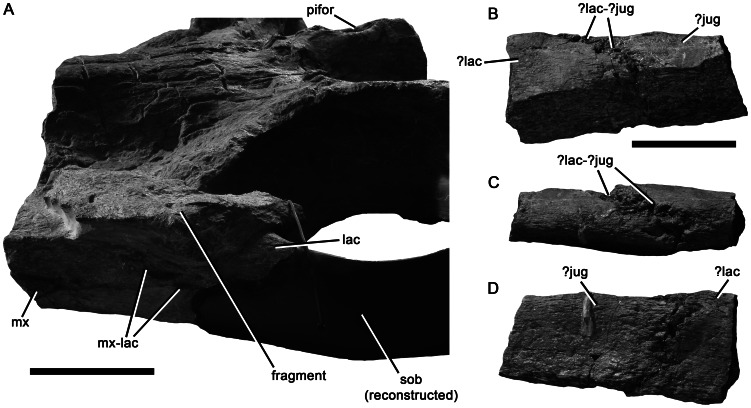
Context of the ‘lacrimal’ of *Pliosaurus kevani* n. sp. DORCM G.13,675. Left orbit and antorbital region in dorsolateral view (A), the unidentified bone fragment attached anterior to the orbit by matrix is not the same as the suborbital bar fragment in (B–D). Portion of possible suborbital bar fragment in medial or lateral (B, D) and dorsal (C) views. Abbreviations: jug, jugal; lac, ‘lacrimal’; ?lac-?jug, possible ‘lacrimal’-jugal contact; mx, maxilla; mx-lac, maxilla-’lacrimal’ contact; pifor, pineal foramen; sob, suborbital bar. Scale bars equal 100 mm (A) and 50 mm (B–D).

**Figure 8:**
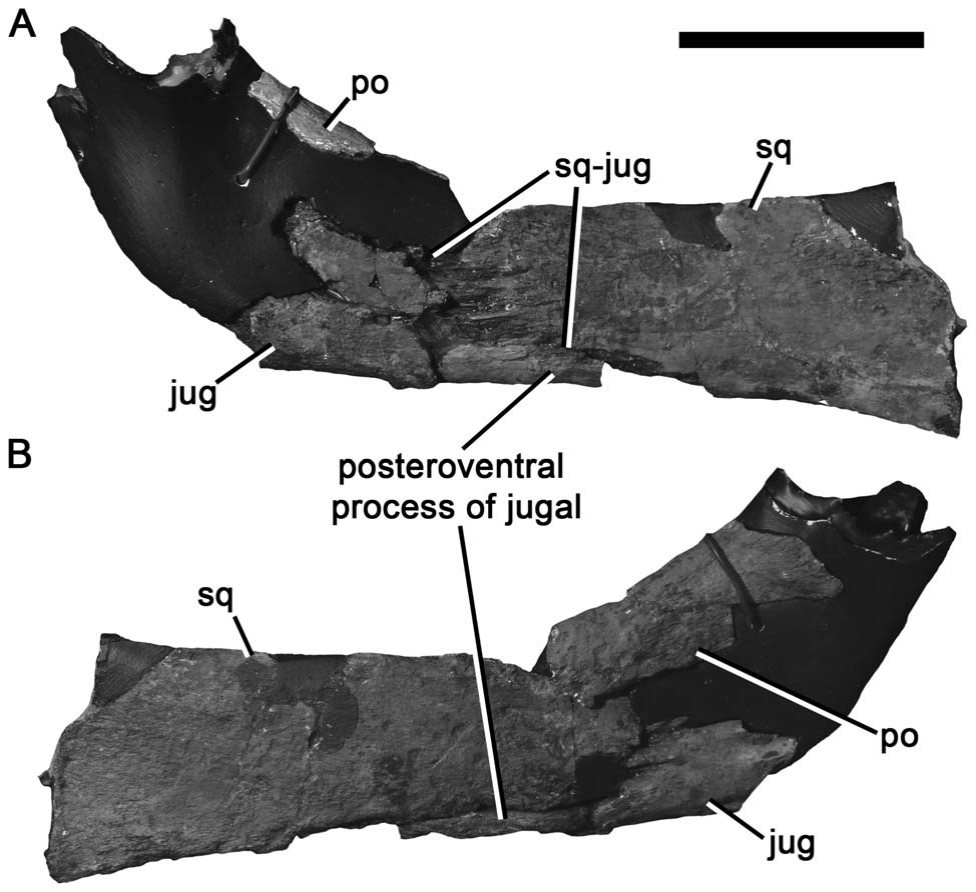
Anterior portion of the right suborbital bar of *Pliosaurus kevani* n. sp. DORCM G.13,675. In medial (A) and lateral (B) views. Abbreviations: jug, jugal; po, postorbital; sq, squamosal; sq-jug, squamosal-jugal contact. Scale bar equals 100 mm.

**Figure 9:**
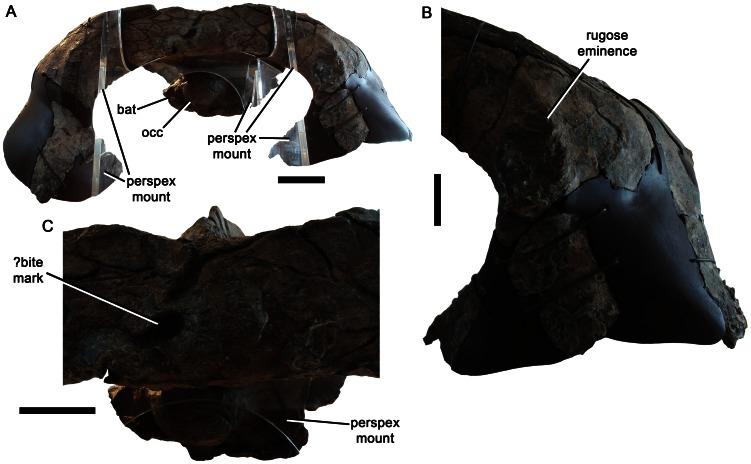
Suspensorium of *Pliosaurus kevani* n. sp. DORCM G.13,675 in posterior view. Posterior view of entire suspensorium (A), of the left ventral ramus (B), and of the squamosal-squamosal contact (C). Abbreviations: bat, basal tuber; occ, occipital condyle. Scales bar equal 100 mm (A) and 50 mm (B–C).

**Figure 10:**
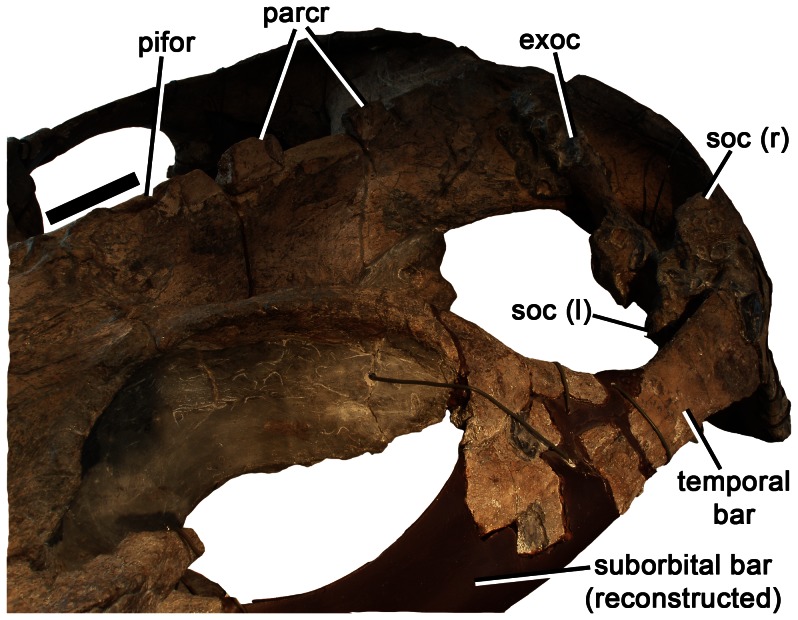
Left orbit and postorbital region of *Pliosaurus kevani* n. sp. DORCM G.13,675 showing parietal crest in anterolateral view. Abbreviations: exoc, exoccipital-opisthotic; pifor, pineal foramen; parcr, parietal crest; soc (l), left portion of supraoccipital; soc (r), right portion of supraoccipital. Scale bar equals 100 mm.

**Figure 11:**
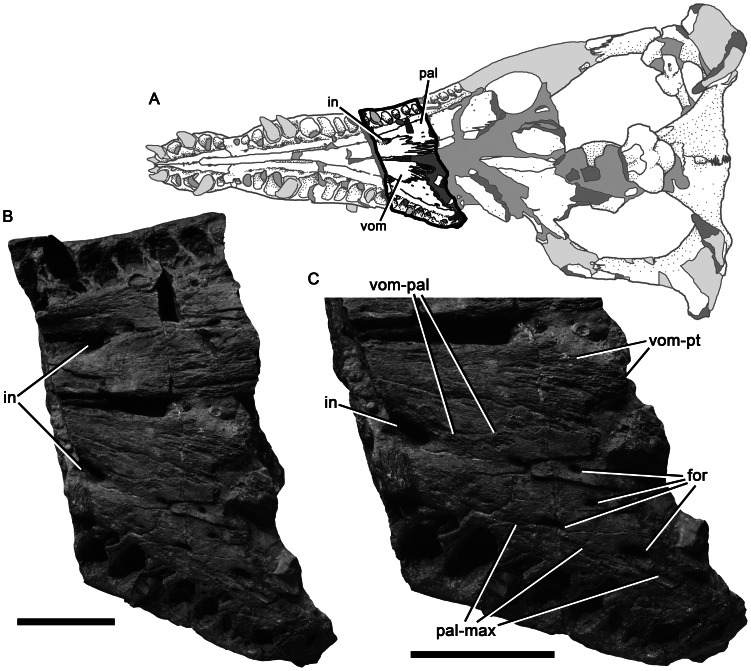
Palate of *Pliosaurus kevani* n. sp. DORCM G.13,675 in the region of the internal naris, seen in ventral view. Schematic (A) showing the portion of the palate figured in (B–C). (C) is at greater magnification than (B). Abbreviations: for, foramina; in, internal naris; pal, palatine; pal-max, palatine-maxilla contact; vom, vomer; vom-pal, vomer-palatine contact; vom-pt, vomer-pterygoid contact. Scale bars equal 100 mm.

**Figure 12:**
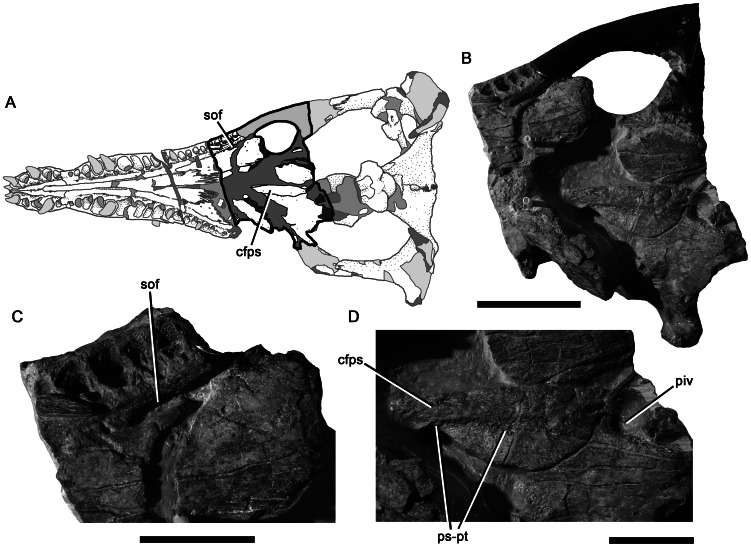
Posterior palate of *Pliosaurus kevani* n. sp. DORCM G.13,675. Schematic (A) showing the portion of the palate figured in (B–D). Posterior portion of palate in ventral view with various regions shown at magnification (B–D). Abbreviations: cfps, cultriform process of the parasphenoid; piv, posterior interpterygoid vacuity; ps-pt, parasphenoid-pterygoid contact; sof, suborbital fenestra. Scale bars equal 200 mm (B) and 100 mm (C–D).

**Figure 13:**
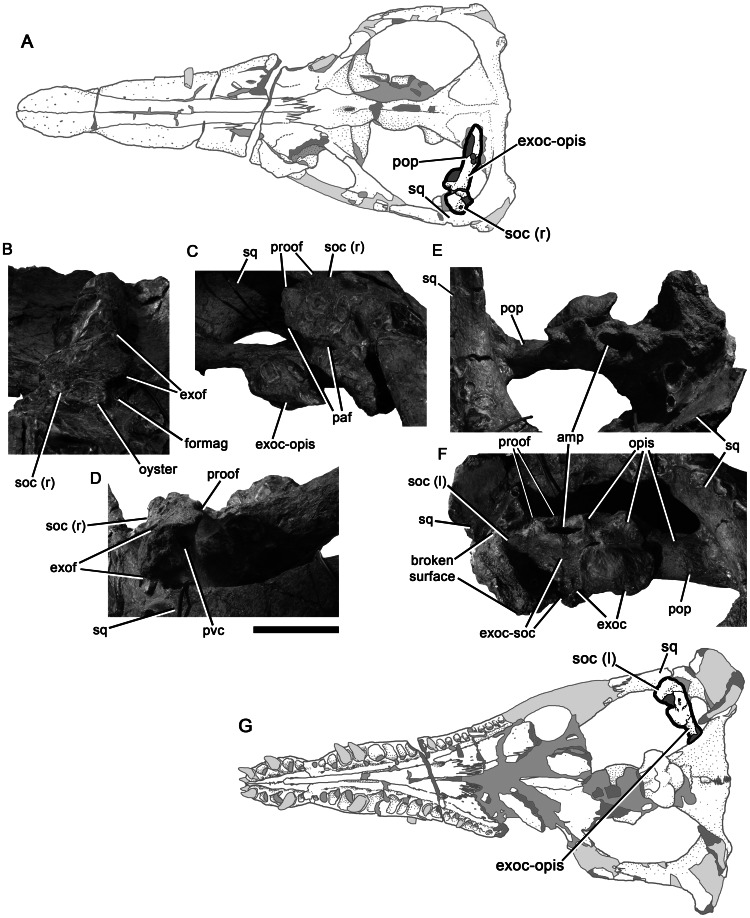
Bones of the otic capsule of *Pliosaurus kevani* n. sp. DORCM G.13,675. Schematic (A) showing the portions of the otic capsule figured in (B–D). Right portion of the supraoccipital in posterior (B), right ventrolateral (C) and right posterolateral (D) views. Schematic (G) showing the portions of the otic capsule figured in (E–F). Left exoccipital-opisthotic and articulated left portion of the supraoccipital in anteromedial (E) and ventral (F) views. Abbreviations: amp, ampullary recess in opisthotic; exoc, exoccipital; exoc-opis, exoccipital-opisthotic; exoc-soc, exoccipital-supraoccipital contact; exof, exoccipital facet of the supraoccipital; formag, supraoccipital portion of the foramen magnum; opis, opisthotic; paf, parietal facet of the supraoccipital; pop, parocipital process of the opisthotic; proof, prootic facet; pvc, posterior vertical canal; soc (l), left portion of the supraoccipital; soc (r), right portion of the supraoccipital; sq, squamosal. Scale bar equals 100 mm.

**Figure 14:**
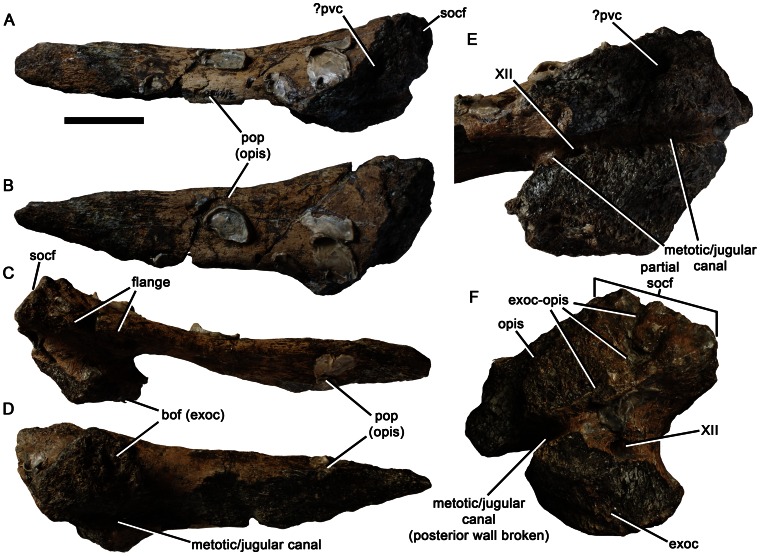
Right exoccipital-opisthotic of *Pliosaurus kevani* n. sp. DORCM G.13,675. In anterior (A), dorsal (B), posterior (C), ventral (D), anteroventral (E), and medial (F) views. Abbreviations: bof (exoc), exoccipital portion of the basioccipital facet; exoc, exoccipital; exoc-opis, exoccipital-opisthotic suture; opis, opisthotic; pop (opis), paroccipital process; pvc, posterior vertical canal; socf, supraoccipital facet of the exoccipital-opisthotic; XII, canal for the hypoglossal nerve opening into the metotic/jugular canal. Scale bar equals 50 mm (A–D) or 25 mm (E–F).

**Figure 15:**
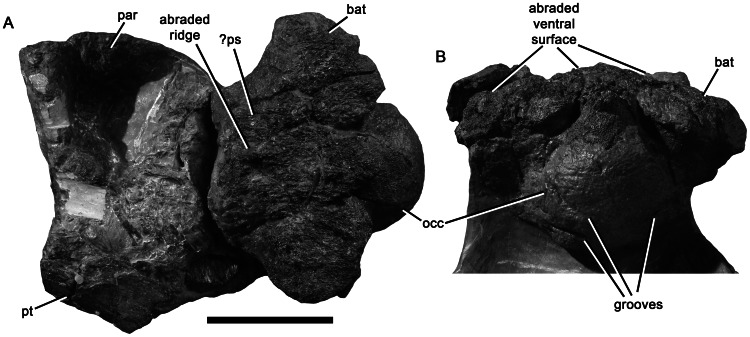
Posterior part of the basicranium of *Pliosaurus kevani* n. sp. DORCM G.13,675. In ventral (A) and posterior (B) views. Abbreviations: bat, basal tuber; occ, occipital condyle; par, parietal; ps, parasphenoid; pt, pterygoid. Scale bar equals 100 mm.

**Figure 16:**
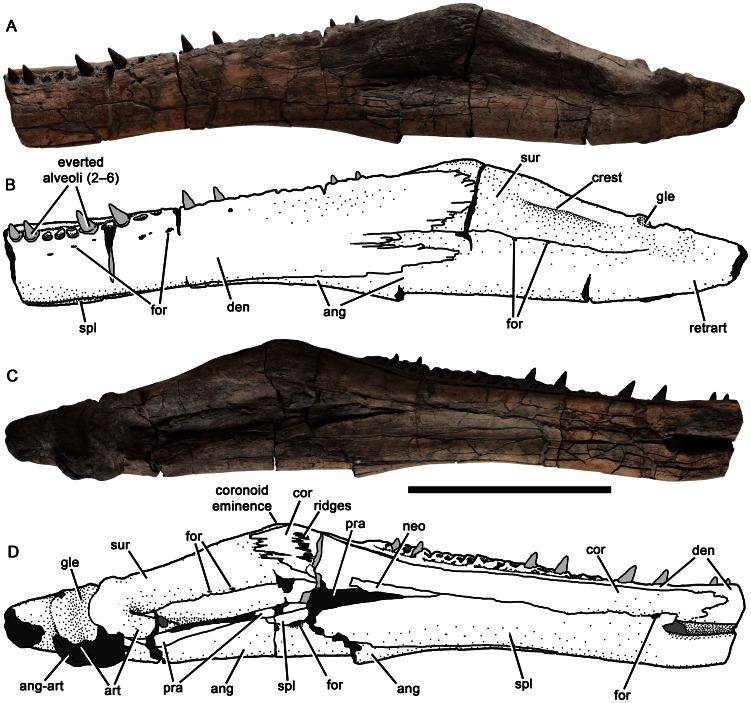
Left mandible of *Pliosaurus kevani* n. sp. DORCM G.13,675. In lateral (A–B) and medial (C–D) views. In line drawings (B, D) dark grey tone represents broken bone surface, mid grey represents matrix, and light grey represents tooth or artificial restoration. Abbreviations: ang, angular; ang-art, angular-articular contact; art, articular; cor, coronoid; den, dentary; for, foramina or foramen; gle, glenoid; neo, possible neomorphic ossification; pra, prearticular; retrart, retroarticular process; spl, splenial; sur, surangular. Scale bar equals 500 mm.

**Figure 17:**
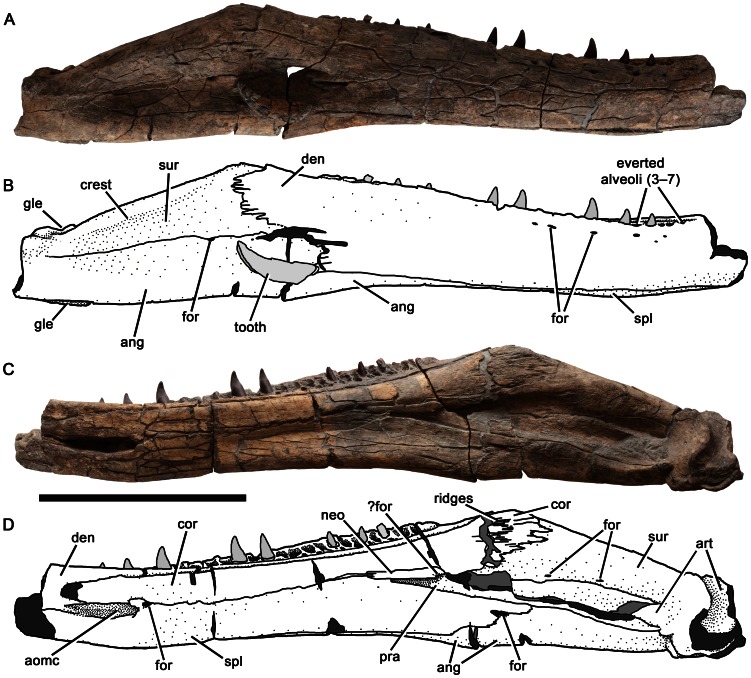
Right mandible of *Pliosaurus kevani* n. sp. DORCM G.13,675. In lateral (A–B) and medial (C–D) views. In line drawings (B, D) dark grey tone represents broken bone surface, mid grey represents matrix, and light grey represents tooth or artificial restoration. Abbreviations: ang, angular; aomc, anterior opening of Meckel's canal; art, articular; cor, coronoid; den, dentary; for, foramina or foramen; gle, glenoid; neo, possible neomorphic ossification; pra, prearticular; spl, splenial; sur, surangular. Scale bar equals 500 mm.

**Figure 18:**
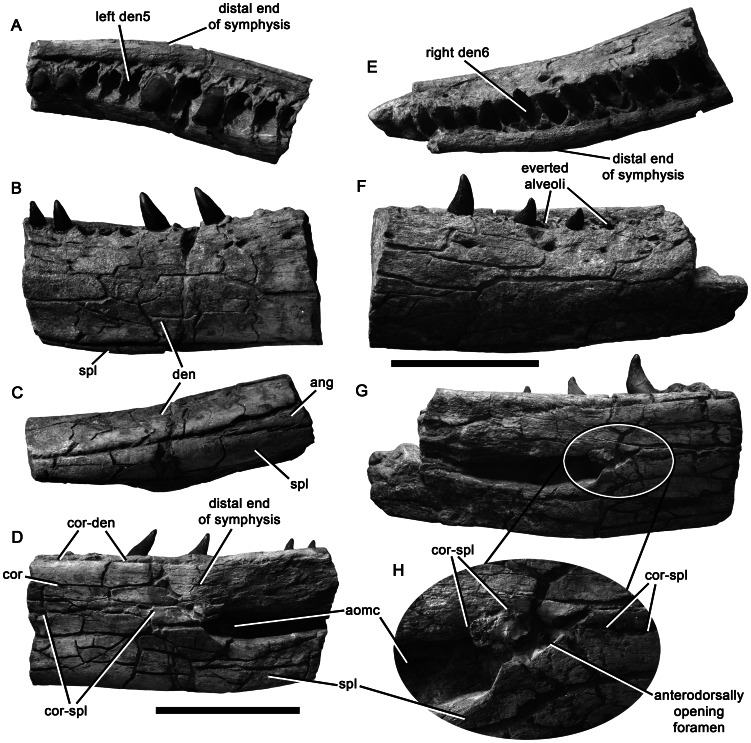
Anterior preserved portions of the mandibles of *Pliosaurus kevani* n. sp. DORCM G.13,675. Anterior portion of left mandible in dorsal (A), lateral (B), ventral (C), and medial (D) views. Anterior portion of right mandible in dorsal (E), lateral (F), and medial (G–H) views with magnification (x2.0) of the region posterior to the anterior opening of Meckel's canal (H). Abbreviations: ang, angular; aomc, anterior opening of Meckel's canal; cor, coronoid; cor-den, coronoid-dentary contact; cor-spl, coronoid-splenial contact; den, dentary; den5, fifth preserved dentary alveolus; den6, sixth preserved dentary alveolus; spl, splenial. Scale bar equals 200 mm.

**Figure 19:**
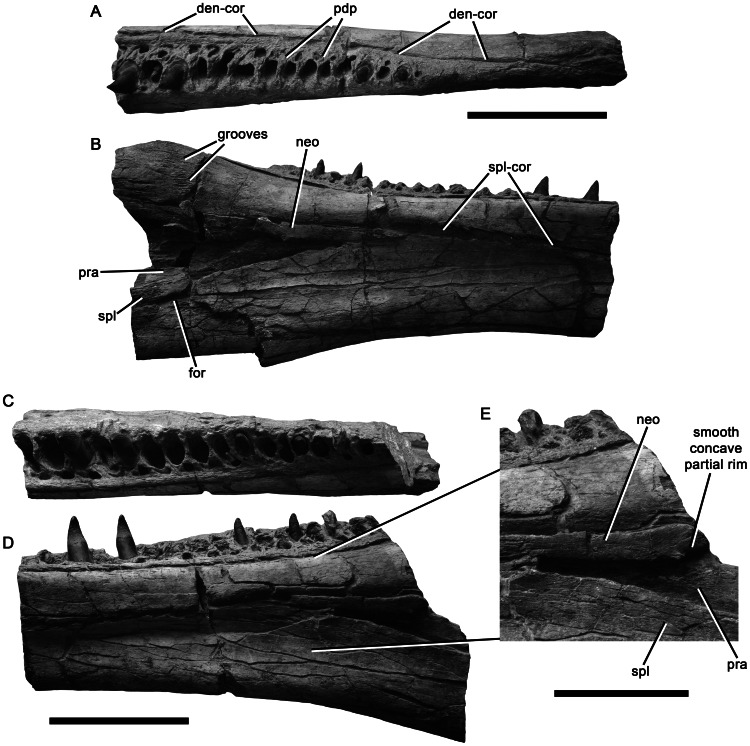
Central portions of the mandibles of *Pliosaurus kevani* n. sp. DORCM G.13,675. Central portion of left mandible in dorsal (A) and medial (B) views. Central portion of right mandible in dorsal (C) and medial (D–E) views, showing an enlarged view of the possibly neomorphic ossification (E). Abbreviations: den-cor, dentary-coronoid contact; for, foramen; neo, possibly neomorphic ossification; pdp, paradental plates; pra, prearticular; spl, splenial; spl-cor, splenial-coronoid contact. Scale bars equal 200 mm (A–D) and 100 mm (E).

**Figure 20:**
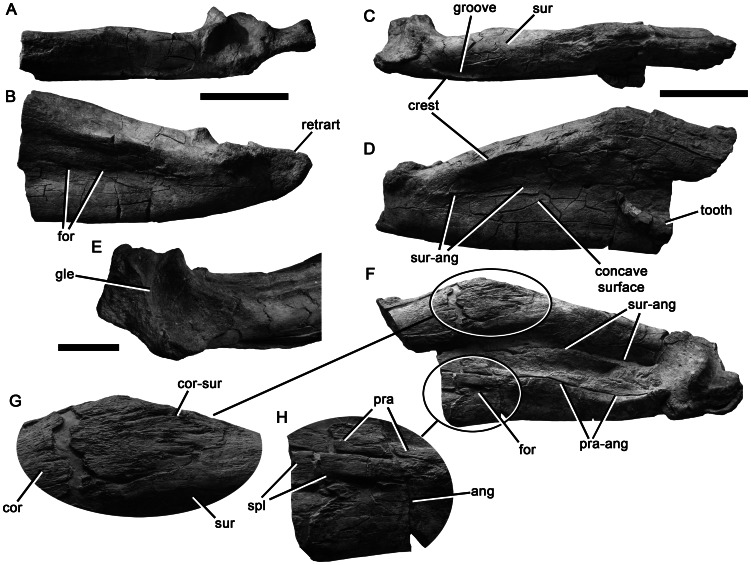
Posterior portions of the mandibles of *Pliosaurus kevani* n. sp. DORCM G.13,675. Posterior portion of the left mandible in dorsal (A) and lateral (B) views. Posterior portion of the right mandible in dorsal (C), lateral (D), posterodorsomedial (E), and medial (F, G, H) views with magnifications (x2.0) of the coronoid-surangular contact (G) and foramen at the splenial-angular contact (H). Abbreviations: ang, angular; cor, coronoid; cor-sur, coronoid-surangular contact; for, foramen or foramina; gle, glenoid; pra, prearticular; pra-ang, prearticular-angular contact; retrart, retroarticular process; spl, splenial; sur, surangular; sur-ang, surangular-angular contact. Scale bars equal 200 mm (A–D, F) and 100 mm (E).

**Figure 21:**
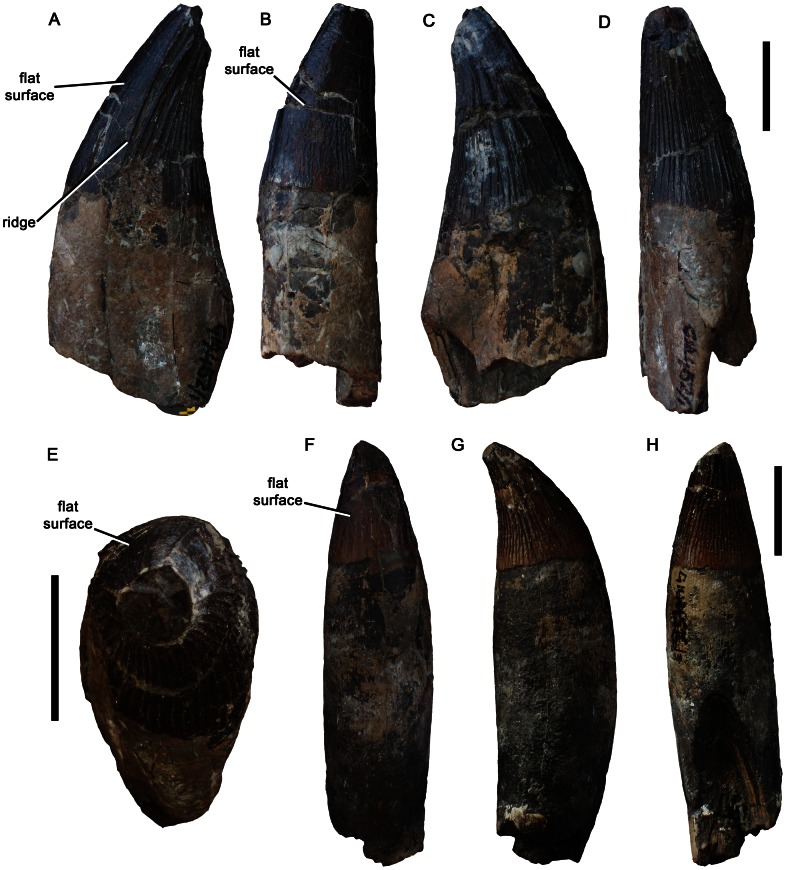
Teeth of *Pliosaurus kevani* n. sp. DORCM G.13,675. Large tooth in mesial or distal (A, C), labial (B), lingual (D), and apical (E) views. Small tooth in labial (F), mesial or distal (G), and lingual (H) views. Scale bars equal 20 mm.

**Figure 22:**
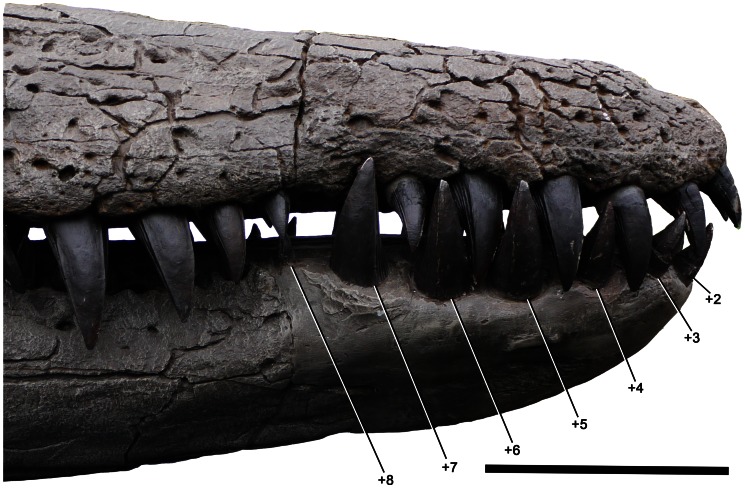
Hypothetical reconstruction of the right symphysial dentition of *Pliosaurus kevani* n. sp. DORCM G.13,675. Eight additional alveoli are reconstructed based on the estimated missing portion, and pattern of interlock with the preserved cranial teeth. The reconstructed alveoli are marked ‘+2’, ‘+3’ etc. A small mesialmost alveolus is not visible in lateral view. Scale bar equals 200 mm.

#### Etymology

Species named after Kevan Sheehan, the main collector of DORCM G.13,675. The name also serves as a tribute to the underestimated and undervalued Kevans of this world.

#### Locality and horizon

Wyke Siltstone bed (*Rasenia cymodoce* Zone, Lower Kimmeridgian), Kimmeridge Clay Formation, Ancholme Group of Osmington Bay (UK Ordnance Survey grid reference SY 372520 81930; Global Positioning System WGS84: 50° 38′ 11″ N 2° 23′ 23″ W), Dorset, United Kingdom (Fig. 1). The stratigraphic section at Black Head was given by ([63]:fig. 6).

#### Tentatively referred specimen

CAMSM J.35990 is most of a postcranial skeleton, originally referred to *Stretosaurus macromerus* [17], [56]–[57]. It was found at Stretham, southwest of Ely in Cambridgeshire, probably from the Lower Kimmeridgian *Aulacostephanus mutabilis* Zone [34]. This specimen is significant because relatively complete postcranial data are available, although only fragments of the skull remain. CAMSM J.35990 differs from most specimens of *Pliosaurus* in possessing subtrihedral teeth, which are otherwise present only definitely in *Pliosaurus kevani* n. sp., and possibly also in *Gallardosaurus iturraldei* from the Oxfordian of Cuba (M.E. pers. obs.; see below). Because of the paucity of preserved postcrania in several other species of *Pliosaurus*, especially *P. kevani*, which is known only from a skull, CAMSM J.35990 cannot be confidently diagnosed as a distinct species, or referred to an existing species with certainty. However, we provisionally refer it to *Pliosaurus* cf. *kevani* based on the presence of subtrihedral teeth and very large body size.

A subtrihedral tooth from the Kimmeridge Clay Formation of Ely, Cambridgeshire, UK is also referred to *Pliosaurus* cf. *kevani* (LEICT (New Walk Museum and Art Gallery, Leicester, United Kingdom) G418.1965.108).

#### Diagnosis

Species of *Pliosaurus* with four autapomorphies, which are absent in all other species of *Pliosaurus*: (1) subrectangular sheet of the maxilla extends anteriorly on alveolar surface of the premaxilla to contact the distalmost premaxillary alveolus — in other species of *Pliosaurus* an interdigitating premaxilla-maxilla suture is located mid-way between the mesialmost maxillary and distalmost premaxillary alveoli; (2) pineal foramen surrounded by a raised rim — in other thalassophonean pliosaurids, including other species of *Pliosaurus*, a shallow fossa containing anteroposteriorly oriented grooves/ridges extends anteriorly from the pineal foramen; (3) mesial postsymphysial dentary alveoli everted to face dorsolaterally — in other species the dentary alveoli all face dorsally; (4) lateral surface of the mandible dorsoventrally concave posteriorly — in other thalassophonean pliosaurids the lateral surface of the postedentary bones is flat or weakly convex. *P. kevani* also possesses the following unique character combination: high dentary alveolar count including 22 postsymphysial alveoli (>28 total) and an estimated total count of 36–37; high count of symphysial dentary alveoli (>6), estimated as 14–15; teeth subtrihedral, possessing a suboval cross-section with only a slightly flattened labial surface bearing only sparse enamel ridges; pronounced mediolateral expansion of caniniform regions of the premaxilla and maxilla; six closely-spaced premaxillary alveoli; distalmost premaxillary alveolus reduced compared to more mesial alveoli (i.e. anisodont [ = ’heterodont’] premaxillary dentition); premaxilla–parietal suture located level with the anterior region of the orbit. Because only the skull of *P. kevani* is known, the condition of postcranial characters that vary among other species of *Pliosaurus* cannot be determined.

#### Remark

*Pliosaurus kevani* is described in detail later in this paper.

### *Pliosaurus westburyensis* n. sp

urn:lsid:zoobank.org:act:DF34CD25-6F48-4C08-ACFF-D617FA81F5C6

1993 *Pliosaurus brachyspondylus* Owen; Taylor & Cruickshank 1993 ([54]:p. 401, figs 3–11)

2012 *Pliosaurus* sp.; Sassoon et al. 2012 ([55]:p. 769, figs 19D–F)

2012 *Pliosaurus* sp.; Knutsen 2012 ([34]:p. 265, figs 2A, 4D, 5E–F)

#### Holotype

BRSMG (Bristol City Museum and Art Gallery, Bristol, United Kingdom) Cc332, a skull and postcranial fragments.

#### Etymology

Species named after the town of Westbury near which BRSMG Cc332 was found.

#### Locality and horizon

Subdivision E5 [64] of the *Aulacostephanus euxodus* Biozone (Upper Kimmeridgian), one metre below the *Crussoliceras* Limestone of the Kimmeridge Clay Formation of Westbury Clay Pit, Wiltshire, United Kingdom.

#### Diagnosis

Species of *Pliosaurus* with three autapomorphies: (1) premaxillary alveoli widely spaced, with interalveolar walls approximately half the anteroposterior length of a single alveolus; (2) a long, sheet-like process of the maxilla extends posteromedial to the anterolateral part of the maxilla–frontal contact medial to the external naris. This process of the maxilla terminates just anterior to orbital midlength ([65]:fig. A1); (3) premaxilla–parietal suture located around orbital midlength. *P. westburyensis* also possesses the following unique character combination: low dentary alveolar count including only 18 postsymphysial alveoli (the symphysis is missing so a full count is not possible); teeth fully trihedral, possessing a flat, anteroposteriorly broad labial surface lacking enamel ridges; mediolateral expansion of premaxilla and maxillary caniniform region relatively slight; six premaxillary alveoli; distalmost premaxillary alveolus similar in size to more mesial alveoli (i.e. lacks anisodont premaxillary dentition); space between maxillary and premaxillary alveolar rows comparable to other interalveolar spaces (i.e. diastema absent); cervical centra lacking ventral ridge.

#### Remarks

Sassoon et al. observed several differences between the holotype of *Pliosaurus westburyensis* (BRSMG Cc332 [54]) and that of *P. carpenteri* n. sp. (BRSMG Cd6172) in the snout, parietal crest, and alveolar count [55]. They suggested these differences represented intraspecific variation, with these specimens possibly being sexual dimorphs. However, although these specimens are from close stratigraphic levels of the same quarry, the differences between them are relatively great when seen in the context of specimens from other localities, and warrant specific distinction. In the snout, the wide alveolar spacing of BRSMG Cc332 is unique and is an autapomorphy of *P. westburyensis*. The narrow snout of *P. westburyensis*, which shows relatively little lateral expansion of the canniniform regions of the premaxilla and maxilla, is shared with some species, including *Pliosaurus brachydeirus*, but differs from others including *P. carpenteri* and *P. kevani*. The dorsally high, anteroposteriorly extensive parietal crest of *P. westburyensis* differs from the low crest of *P. carpenteri*, but other *Pliosaurus* specimens do not preserve the crest so comparisons cannot be made.

Sassoon et al. measured the position of the parietal–premaxilla contact as a proportion of skull length in both BRSMG Cc332 and Cd6172 and found they had similar measurements [55]. However, its position compared to other cranial landmarks may be autapomorphic in *P. westburyensis*: the anteriormost point of the parietal–premaxilla contact is posterior to orbital midlength (pers. obs. BRSMG Cc332 [54]). In contrast, the contact extends anterior to orbital midlength in other thalassophoneans [65], including *Pliosaurus kevani* (Figs 2–3), and likely also in *P. carpenteri*, although damage to the orbits and interorbital region obscures the condition slightly in *P. carpenteri*.

Sassoon et al. also stated that BRSMG Cc332 and Cd6172 had different dentary and maxillary alveolar counts [55]. However, both have 18 postsymphysial dentary alveoli (the maxillary alveolar count can only be estimated imprecisely in Cd 6172 [55], and the mandibular symphysis is not preserved in BRSMG Cc332, but seems likely to have contained a similar number of alveoli to that in BRSMG Cd6172, which has nine [55]).

### *Pliosaurus carpenteri* n. sp

urn:lsid:zoobank.org:act:9F12DB6D-EF17-41C8-AB76-8221A854ED8A

2012 *Pliosaurus* sp.; Sassoon et al. 2012 ([55]:p. 746, figs 2–18, 19A–C)

2012 *Pliosaurus* sp.; Knutsen 2012 ([34]:p. 265, fig. 3)

#### Holotype and only specimen

BRSMG Cd6172.

#### Etymology

Species named after Simon Carpenter, who discovered and collected BRSMG Cd6172.

#### Locality and horizon

Subdivision E4 [64] of the *Aulacostephanus euxodus* Biozone (Upper Kimmeridgian), seven metres below the *Crussoliceras* Limestone) of the Kimmeridge Clay Formation of Westbury Clay Pit, Wiltshire, United Kingdom.

#### Diagnosis

Species of *Pliosaurus* possessing a single autapomorphy: the dorsal surface of the surangular lacks any fossa (unlike in thalassophonean pliosaurids other than CAMSM J.35991 [52], the proposed ‘neotype’ of *P. brachyspondylus* [34]), and faces dorsally — in other specimens of *Pliosaurus* it is inclined to face dorsolaterally. *P. carpenteri* also possesses the following unique character combination: low dentary alveolar count including only 18 postsymphysial alveoli, and a total count of 27; intermediate low count of syphysial alveoli (nine); teeth fully trihedral, possessing a flat, anteroposteriorly broad labial surface lacking enamel ridges; mediolateral expansion of caniniform regions of the premaxilla and maxilla relatively pronounced (although this may have been enhanced by ventral crushing); six closely-spaced premaxillary alveoli; distalmost premaxillary alveolus reduced compared to more mesial alveoli (i.e. anisodont premaxillary dentition); diastema present between maxillary and premaxillary alveolar rows; premaxilla–parietal suture located level with the anterior region of the orbit; cervical centra lacking ventral ridge; epipodials with highly convex proximal surfaces.

### The cranium of *Pliosaurus kevani* n. sp

The skull of *Pliosaurus kevani* is large (1995 mm long on the dorsal midline) and longirostrine, with a preorbital portion 1130 mm long, thus comprising 57% of the skull length (Fig. 2). A full cranial reconstruction is shown in Figure 3. The temporal region is transversely broad (730 mm) relative to its length (postorbital length  =  520 mm; temporal fossa length  =  670). The skull has been slightly crushed dorsoventrally, especially immediately anterior to the orbits. The postorbital portion of the skull is rotated slightly dorsally. The snout and dorsal surfaces of the skull are complete. However, the suborbital and subtemporal bars, and the basicranium and palate posterior to the vomer-pterygoid contact, are only partly preserved. Some of the palatal elements have been broken and pulled apart either anteroposteriorly or mediolaterally, and the ventral portions of the squamosal-quadrate unit, which formed the mandibular condyles, are missing.

#### Premaxilla

The body of the premaxilla, which forms the anterior part of the snout, has a tooth-bearing ventral portion that measures 300 mm anteroposteriorly. It is dorsoventrally low and mediolaterally broad (215 mm) (Fig. 4). Six premaxillary alveoli are present, of which the first (mesialmost) alveolus is highly reduced, with a minimum diameter (28 mm) approximately half that of the third alveolus (58 mm). Substantial reduction of the first premaxillary alveolus is a synapomorphy of Late Jurassic and younger pliosaurids (*Pliosaurus* + Brachaucheninae [26]:character 140, [27]), and also occurs in most plesiosauroids (e.g., [66]). However, this alveolus in less reduced in *Pliosaurus* than it is in brachaucheninines [27], so the condition in *Pliosaurus* is tentatively considered to be autapomorphic.

The third–fifth premaxillary alveoli of DORCM G.13,675 are the largest, demonstrating the presence of an anisodont premaxillary dentition (‘heterodont’ is often used to describe this condition in plesiosaurians, but ‘anisodont’ is more appropriate because the teeth vary only in size, not morphology). This is similar to the condition in some Kimmeridge Clay Formation pliosaurids, which, like DORCM G.13,675, have a reduced distalmost premaxillary alveolus (e.g., [55]). However, it differs from others, in which the distalmost premaxillary alveolus is only slightly smaller than more mesial alveoli (e.g., [54]) (Table 1). The premaxilla of DORCM G.13,675 is transversely expanded to accommodate the large third–fifth alveoli. Thus, its outline in dorsal view has pronouncedly convex lateral margins, resulting in a ‘spatulate’ appearance (Figs 2–5), the prominence of which is also variable among Kimmeridge Clay Formation pliosaurids (Table 1). A transversely narrow ‘rostral constriction’ separates the expanded region of the premaxilla from the maxilla (Fig. 2). The premaxillary dentition is separated from the maxillary dentition by a smooth, edentulous region of compact bone forming a diastema subequal to (left) or greater than (right) the diameter of the distalmost premaxillary alveolus. This diastema is formed by a subrectangular, sheet-like anterior extension of the maxilla, which contacts the posterior margin of the distalmost ( = sixth) premaxilary alveolus. This extension of the maxilla is absent in all other pliosaurids (e.g., [16], [23]–[24], [54]–[55]), and is an autapomorphy of *Pliosaurus kevani* n. sp. (Fig. 4). The premaxillary alveoli of DORCM G.13,675 are otherwise closely spaced, divided only by the lateral extensions of rugose, triangular paradental plates. A deep, anteroposteriorly oriented groove separates the paradental plates from the central platform that bears the interdigitating midline contact of the premaxillae. This platform is mediolaterally narrow anteriorly, where it contributes to the posteromedial margin of the first premaxillary alveolus. The platform bifurcates posterior to the fourth alveolus, forming paired posterolateral extensions that contact the maxillae posteriorly. The recess between these posterolateral extensions accommodates the anterior process of the vomer. Three foramina penetrate the premaxillary-vomerine contact: an anterior midline foramen at the level of the fourth premaxillary alveolus, and paired lateral foramina level with the fifth premaxillary alveolus. Smooth channels extend anterolaterally from the lateral foramina, incising the posterolateral extensions of the central platform.

**Table 1:** Selected measurements and observations on Late Jurassic pliosaurid specimens, arranged stratigraphically (older specimens are shown lower in the table).

			Alveolar counts						
	A	B	Premaxilla	Maxilla	Dentary	Symphysial	C	D	E
**Tithonian**									
*Pliosaurus rossicus* PIN 304 and [7]–[9], [40]	?	Trihedral	5	?	?	6	?	?	?
**Upper Kimmeridgian or Lower Tithonian**									
***P. elegans*** ** or ** ***A. autissidorensis*** ** Biozone**									
NHMUK PV OR 39362 and [15]	1730	?	5	>23	>25 (27e)	>7 (9e)	No	Yes	?
**Upper Kimmeridgian**									
***A. autissidorensis*** ** Biozone**									
*Pliosaurus* indet. SEKC.K1.2 and [53]	2000	?	?	?	29e	8	?	?	?
***A. autissidorensis*** ** or ** ***A. euxodus*** ** Biozone**									
*Pliosaurus* ?*rossicus* OXFUM J.10454 and [17], [57]	2875r	?	?	?	30e	6	?	?	?
***A. euxodus*** ** Biozone**									
*Pliosaurus* ?*brachyspondylus* CAMSM J.35991 and [52]	1200* (small)	Trihedral	?	?	29	9	?	?	Flat*
*Pliosaurus carpenteri* BRSMG Cd6172 and [55]	1800	Trihedral	6	>17	27	9	Yes	?	Convex
*Pliosaurus westburyensis* BRSMG Cc332 and [54]	1600	Trihedral	>4	25	>20	>2	No	No	?
***A. mutabilis*** ** or ** ***A. euxodus*** ** Biozone**									
*Pliosaurus* ‘*portentificus*’ CAMSM J.46380 and [43]	? (big)	?	?	?	>12	8	?	?	?
***A. mutabilis*** ** Biozone**									
*Pliosaurus* cf. *kevani* CAMSM J.35990 and [56]–[57]	? (big)	Subtrihedral	?	?	?	?	?	?	Convex
**Lower Kimmeridgian**									
***Rasenia cymodoce*** ** Biozone**									
*Pliosaurus* indet. BHN 2R.370 and [36]	2050	?	?	?	29	11	?	?	?
*Pliosaurus brachydeirus* OXFUM J.9245	1320i*	Trihedral	>4	>16	>35 (36e)	>11 (12e)	No	?	Flat*
*Pliosaurus kevani* DORCM G.13,675	2045	Subtrihedral	6	>20	> 28 (37e)	>6 (15e)	Yes	Yes	?
**Oxfordian**									
*Gallardosaurus iturraldei* MNHNCu P3005 and [12]	? (small)*	?Subtrihedral	?	?	?	?	?	No	?

Stratigraphic data is from [34]. Abbreviations: e, estimated; r, measurement based on reconstruction; *, measurement or observation of a juvenile specimen. Columns contain data on: A, mandible length; B, tooth morphology; C, presence of anisodont premaxillary dentition (reduced distal alveolus); D, presence of a ventral longitudinal ridge on the parasphenoid; E, morphology of the proximal surface of the radius or tibia. Measurements are in millimetres (mm).

The dorsal and lateral surfaces of the premaxillae are highly fractured, conferring an artefactual rugose appearance (Fig. 4). They bear numerous foramina, especially anteriorly and laterally. On the dorsal surface, the premaxillary midline suture is weakly sinuous anteriorly, but becomes straight posterior to the rostral constriction. The dorsal surface of the snout is mediolaterally convex, except where it has been crushed ventrally, posteriorly. This crushing has preferentially affected the maxillae, causing them to be depressed either side of the posterodorsal processes of the premaxilla, resulting in the appearance of a prominent, anteroposteriorly oriented midline ridge. However, this is artefactual: in fact no dorsomedian ridge was originally present. The lateral margins of the posterodorsal processes of the premaxillae form straight, continuous lines that extend posterodorsally, separating the premaxillae from the maxillae anteriorly (anterior to the external naris) and from the frontals posteriorly. The conjoined posterodorsal processes of the premaxillae extend far posteriorly, forming a broad, deeply interdigitating contact with the parietals adjacent to the anterior orbit margin. Because the premaxillae contact the parietals, the frontals are excluded from the midline in dorsal view (Figs 2–3), as in other thalassophonean pliosaurids and derived members of Rhomaleosauridae, Leptocleidia and Elasmosauridae (e.g., [16], [65], [67]–[69]). However, among these taxa, pliosaurids are unique in possessing a posterior termination of the premaxilla that is mediolaterally broad and interdigitating (*contra* [13], who mistakenly said that this also occurred in cryptoclidids), and they differ from most other taxa in the anterior position of the premaxilla–parietal contact, located anterior to orbital midlength [65], as in DORCM G.13,675. This suture is apomorphically located further posteriorly in *Pliosaurus westburyensis* (Table 1) ([54]:fig. 4).

#### Maxilla

The maxillae form the lateral surfaces of the snout (Figs 2–3, 5). They continue posteriorly in the suborbital region, ventral to the ‘lacrimal’ and jugal. However, because this region is broken, their posterior extent cannot be determined. The left maxilla, as preserved up to the anterior orbit margin, bears 20 alveoli, and the right bears 19 because it is slightly less complete (Fig. 5). The maxillary dentition is anisodont; for example, the mediolateral diameter of the first (mesialmost) maxillary alveolus (27 mm) is approximately half the diameter of the second (51 mm). The body of the maxilla is expanded laterally to accommodate the fourth–sixth alveoli, which are the largest (Figs 5–6). Posterior to these, successive maxillary alveoli are smaller. As in the premaxilla, the medial walls of the maxillary alveoli are defined by rugose, subtriangular paradental plates. An anteroposteriorly oriented groove containing replacement alveoli separates these plates from the horizontal palatal shelf of the maxilla, which contacts the lateral elements of the palate (vomer and palatine) medially. Several irregularly distributed foramina of varying sizes penetrate the maxilla-vomer and maxilla-palatine contacts (Figs 5–6; these two sutures form a continuous line parallel to the tooth row). The internal naris is identified as the largest of these foramina, and is located at the intersection of all three bones, at the level of the eleventh maxillary alveolus. In *Pliosaurus westburyensis* and *Pliosaurus carpenteri* the internal naris is located at the level of the ninth maxillary alveolus (BRSMG Cd6172 [55] and BRSMG Cc332, pers. obs.; *contra* [54]), and in NHMUK PV OR 39362 it is located at the level of the seventh maxillary alveolus. The presence of additional foramina on the maxilla–vomer and maxilla–palatine sutures, anterior and posterior to the internal naris, is unique to thalassophonean pliosaurids among Plesiosauria [24], [54] ([26]:character 69).

The maxilla–premaxilla suture of DORCM G.13,675 is expressed on the ventral and external (‘external’  =  dorsal and lateral) surfaces of the snout. Externally the suture originates at the level of the rostral constriction, where it is deeply interdigitating, with a ‘zig-zag’ appearance in lateral view (Fig. 4), and trends posterodorsally. The presence of a deeply interdigitating anterior portion of the premaxilla–maxilla suture is a unique synapomorphy of *Pliosaurus* ([26]:character 24). Posterior to this, the premaxilla–maxilla suture becomes weakly sinuous, and the medial edge of the maxilla dorsally overlaps the premaxilla (Fig. 6). This overlap has been accentuated by ventral crushing of the snout, especially in the posterior half of the preorbital region. A posteromedial extension of the maxilla extends medial to the external naris, and contacts an anterolateral extension of the frontal, thus excluding the premaxilla from the external naris (Figs 2–3), as occurs in pliosaurids and leptocleidians (e.g., [5], [16], [22], [45], [67], [70]). The maxilla–frontal contact of DORCM G.13,675 is deeply interdigitating and trends medially. The posteromedial extension of the maxilla is divided into three prong-like processes by anteroposteriorly oriented fissures, which are most clearly visible on the left side (Fig. 6). They are somewhat obscured by damage on the right side, but at least two such processes are clearly present there (Fig. 6). These processes terminate posteriorly around one-quarter of the length of the external naris. Fissures dividing the posteromedial process of the maxilla into prong-like processes are also present in the well-preserved skull of the holotype of *Pliosaurus westburyensis* (BRSMG Cc 332) ([65]:fig. A1), and in brachauchenines (e.g., National Museum of Natural History, Smithsonian Institution, Washington D.C., USA 2361 ([65]:fig. A2); Queensland Museum, Brisbane, Australia (QM) F51291). However, in brachaucheninines the posteromedial process of the maxilla extends posteriorly past the external naris ([27], [65]:character 15), unlike in most Jurassic pliosaurids, including DORCM G.13,675. In *P. westburyensis*, only the most medial prong of the posteromedial process extends posteriorly past the naris ([65]:fig. A1).

The external nares are relatively large and slightly dorsoventrally crushed, oval openings, anteroposteriorly long (left, 116 mm; right, 118 mm) and mediolaterally narrow (left, 38.5 mm; right 24 mm). They are located slightly posterior to the level of the internal nares, as in some other large-skulled plesiosaurians (e.g., [71]).

#### ‘Lacrimal’

The presence of a neomorphic ossification forming the anteroventral margin of the orbit, and informally termed the ‘lacrimal’ [5], [16], [72], is a unique synapomorphy of Pliosauridae [22], [24], [65], [68] ([67] and [73] observed the same morphology but interpreted it as an anterior extension of the jugal). The homology of this element is uncertain, but because the lacrimal is primitively absent in plesiosaurs and other sauropterygians, the ossification in pliosaurids is probably neomorphic and not a direct homologue of the lacrimal of other tetrapods. In DORCM G.13,675 the anterior margin of the ‘lacrimal’ (i.e. the ‘lacrimal’-maxilla suture) is visible on both sides of the skull (Fig. 7A), and its morphology shows that the maxilla continues ventrally under the ‘lacrimal’, forming the alveolar margin of the cranium (Figs 3, 5). Because the suborbital bar is broken on both sides of the skull, much of this region cannot be observed. However, a preserved bone fragment may represent the dorsal portion of one of the suborbital bars (Fig. 7B–D). This fragment bears a strongly interdigitating, subvertical suture that may represent the ‘lacrimal’-jugal contact, which is located at approximately orbital midlength in other pliosaurids [5], [16], [24], [72]. Because of its ‘bar-like’ morphology, this bone fragment must have formed part of either the suborbital or subtemporal bar. Because the subtemporal bars are preserved articulated, our interpretation of the fragment as part of the suborbital bar is most plausible.

#### Prefrontal

The interorbital skull roof is abraded, but some sutures are visible, allowing recognition of an ossification identified as the prefrontal, and possibly a ‘palpebral’ ossification on the lateral surface of the prefrontal. This region has a convex lateral margin that embays the anterodorsal orbit margin (Figs 2–3; its prominence has been reduced by abrasion), as occurs in other thalassophonean pliosaurids and leptocleidids [5], [16], [26], [54], [70], [74]. A similar embayment of the orbital margin, attributed to the frontal, has been described in well-preserved polycotylid skulls [75] ([67]:character 24). In the pliosaurid *Peloneustes*, this projection into the orbit is formed by a separate ossification on the lateral surface of the prefrontal, informally termed the ‘palpebral’ [24]. However, the palpebral-prefrontal suture is only visible in subadults and juveniles (it is closed in adults). Due to abrasion in DORCM G.13,675, it is difficult to determine the presence or absence of this suture, but the gross morphological similarity of this region in DORCM G.13,675, *Peloneustes* and other thalassophonean pliosaurids suggests homology.

The prefrontal contacts the ‘lacrimal’ anteroventrally, around orbital midheight, in a subhorizontal suture that is partly obscured by a break between preserved skull portions on the right side, and concealed by a disarticulated bone fragment on the left side. Part of the prefrontal-maxilla contact is also recognisable, indicating that the prefrontal did not extend anteriorly to contact the external naris, unlike in many plesiosauroids, brachaucheninine pliosaurids [5], [27] and possibly *Liopleurodon* [68].

#### Frontal

Because of poor preservation, many sutures of the frontal could not be recognised. However, the preserved morphology suggests that the exposure of the frontal on the dorsal surface of the skull is anteroposteriorly elongate (Fig. 2), bounded medially by the premaxilla, posteriorly by the parietal and postfrontal, laterally by the prefrontal, and anterolaterally by the maxilla, as in other pliosaurids (e.g., [16], [24], [27], [54], [72]). We could not determine whether the frontal extended laterally between the prefrontal and postfrontal, thus contributing to the dorsal margin of the orbit, or was excluded from the orbit by prefrontal-postfrontal contact.

#### Postfrontal

The postfrontal forms the dorsal portion of the postorbital bar (Fig. 2). It contacts the frontal anteromedially, parietal medially, and postorbital ventrolaterally. The postfrontal-postorbital suture on the lateral surface of the postorbital bar extends posteriorly from a point located at approximately two-thirds the dorsoventral height of the orbit. Close to the posterior margin of the postorbital bar, this suture inflects posteroventrally to contact an angular tubercle on the posterior surface of the bar. The postorbital bar is anteroposteriorly narrow in lateral view. It extends medially as a broad, anterodorsally inclined sheet that forms the anterior wall of the temporal fossa (and posterior wall of the orbital cavity), and contacts the parietal medially (Fig. 2).

#### Postorbital

The left postorbital is almost complete, although its ventral portion is damaged. The postorbital forms the ventral portion of the postorbital bar, and is anteroposteriorly narrow dorsally, but expands ventrally, contacting the jugal (anteroventrally) and squamosal (posteroventrally) (Fig. 2). The postorbital-jugal and postorbital-squamosal sutures form a continuous, non-interdigitating contact, which has a ventrally convex trace in lateral view. This suture originates at the posteroventral margin of the orbit and continues a short distance posterior to the postorbital bar, defining the ventral margin of the short posteroventral process of the postorbital (Figs 2, 8).

#### Jugal

Because both suborbital bars are damaged, the anterior portion of the jugal is incompletely known (although its anterior contact with the ‘lacrimal’ may be preserved in a bone fragment described above; Fig. 7B–D). The posterior portion of each jugal is preserved in articulation with the squamosal (posteriorly) and postorbital (dorsally) (Figs 2, 8). The ventral surface of the posterior half of the jugal is well preserved and smooth, lacking an articular surface for the maxilla. This indicates that the maxilla terminated anterior to this preserved region of the jugal, at the level of the postorbital bar or more anteriorly. A maxilla-squamosal contact was clearly thus absent.

The jugal-squamosal contact is deeply interdigitating (Fig. 8). It is subvertical dorsally, where it originates just posterior to the level of the postorbital bar. From here it curves posteroventrally, defining the dorsal margin of a prominent, ‘prong-like’ posteroventral process of the jugal, which forms most of the ventral surface of the subtemporal bar (Fig. 8). This process is absent in most other pliosaurids, including *Peloneustes* [24] and *Brachauchenius* [27]. However, it is present in *Pliosaurus westburyensis* (BRSMG Cc332), although it was not figured in [54]. The presence of a long posteroventral process of the jugal may be an autapomorphy of *Pliosaurus*, although its presence cannot be determined in many specimens.

#### Squamosal

The squamosal is a triradiate bone (Figs 2–3, 5). It comprises an anterior ramus that contacts the jugal and forms most of the temporal bar, a ventral ramus, which articulates with the quadrate, and a dorsomedial ramus that contacts the midline and forms the posterior margin of the temporal fossa, as in all plesiosaurians (e.g., [76]–[78]). The midline suture of the dorsomedial rami is either closed dorsally, or difficult to observe due to damage. However, it is visible ventrally, where it is deeply interdigitating mediolaterally (Fig. 2). The cross section of the anterior ramus of the squamosal ( = temporal bar) is mediolaterally narrow and dorsoventrally broad (82 mm), less than half the height of the orbit as preserved, and substantially less than that if dorsoventral crushing of the orbit is accounted for. The subtemporal bar has a rounded ventral surface, but a sharp dorsal surface. In lateral view, the subtemporal bar arches dorsally above the level of the maxillary tooth row and mandibular glenoid, as in non-xenopsarian plesiosaurians. This is evident from our reconstruction (Fig. 3). However, it is not immediately apparent when studying the specimen because dorsoventral crushing has obscured the morphology (Fig. 1).

The cross section of the dorsomedial ramus of the squamosal is anteroposteriorly narrow and dorsoventrally broad for most of its length (Fig. 2), as in other non-brachaucheninine thalassophoneans [5], [16], [24], [26]–[27]. It becomes anteroposteriorly thicker, and dorsoventrally lower at its contact with the parietal. The squamosal–parietal contact is complex. Each squamosal forms a thin, anteriorly directed sheet that overlaps the dorsolateral surface of the parietal. Each squamosal also forms a ventral sheet that underlaps the parietal (Fig. 5). Thus, the posterior portion of the parietal is enclosed both dorsally and ventrally by the squamosal and only the parietal crest is exposed on the dorsal surface, and a small rugose midline eminence of the parietal is exposed on the ventral surface (Figs. 3, 5).

The posterior surface of the conjoined squamosals forms a mediolaterally broad convexity that projects posteriorly (Fig. 2). This differs from the mediolaterally narrow, but prominent ‘squamosal bulb’ of many plesiosaurians (e.g., [67]–[68]), including some pliosaurids such as *Thalassiodracon*, *Hauffiosaurus* and *Peloneustes* (e.g., [13], [16], [22]). However, it is similar to the condition in other Late Jurassic pliosaurids and brachaucheninines (BRSMG Cc332, Cd6172 pers. obs. and [5], [9], [12], [27]). A pronounced, irregular depression on the posterior surface in this region of DORCM G.13,675 could be a pathology, a bite mark, or a bone surface degraded during biostratinomy (Fig. 9).

The ventral ramus of the squamosal bears the dorsal portion of the quadrate and is broken ventrally. Because of encrusting organisms and possible sutural fusion, the locations of sutures between the squamosal, quadrate and pterygoid cannot be determined. The posterior surface of the ventral ramus of each squamosal bears a mound-like, rugose eminence, bounded dorsolaterally by a slight ridge that extends dorsomedially along the posterior surface of the squamosal arch (Figs 2, 9). The ventral half of the posterior surface of the quadrate is vertical and curves anterodorsally.

#### Parietal

The parietal forms the central portion of the temporal region, contacting the interorbital skull roof anteriorly, and the dorsomedial rami of the squamosals posteriorly (Fig. 2). As in many Late Jurassic and younger plesiosaurians, the parietal midline suture is closed. The pineal foramen is located adjacent to the anterior part of the temporal fossa. It is surrounded by a raised rim and has a suboval outline 57 mm long anteroposteriorly and 23 mm wide mediolaterally (Fig. 10). This differs from the condition in all other thalassophonean pliosaurids, in which only the posterior margin of the pineal foramen has a raised rim, and the anterior margin opens into an anteroposteriorly elongate depression containing longitudinal ridges and grooves [5], [16], [24] ([65]:character 37).

The parietal crest of DORCM G.13,675 extends posteriorly from the pineal foramen. It is transversely narrow and dorsoventrally deep, and rises dorsally past the level of the skull roof (Fig. 10), resulting in a dorsally convex outline in lateral view, as in other thalassophoneans. In the anterior half of the temporal fossa, the parietal is mediolaterally narrowest, extends ventrally to form the dorsolateral walls of the endocranial cavity, and may have formed a ligamentous attachment with the epipterygoid ventrally (which is not preserved) as in other pliosaurids [24], [67]. More posteriorly, the parietal expands mediolaterally to form a roof over the occiput (the occipital condyle is inset anteriorly, far under this roof). The parietal attains maximum mediolateral width (42 mm) posteriorly, equal to more than half of the total skull width (73 mm). This great proportional width (∼0.5 times the skull width or greater) is also present on other Late Jurassic pliosaurids (e.g., BRSMG Cc 332 and [12]), brachaucheninines [27], [75], and convergently in leptocleidians [66], [70], [74]–[76] ([26]:character 49). In other plesiosaurians, including Middle Jurassic pliosaurids, the posterior part of the parietal is narrower (e.g., [16], [24]).

The parietal of DORCM G.13,675 has been crushed ventrally. The epipterygoids and prootics are not preserved, and the supraoccipital is preserved in two pieces in the left temporal fossa (explained below). Thus, the original relationships of these bones with the parietal are difficult to determine. However, a flat, posteroventrally facing surface located posteriorly on the ventral surface of the parietal anterior to the otic region may have articulated with the prootic.

#### Ectopterygoid

A possible fragment of the left ectopterygoid is preserved dorsal to the fragmentary left pterygoid lateral ramus (Fig. 5). The ectopterygoid might therefore have overlapped the pterygoid at least in this region. Sutures defining the right ectopterygoid cannot be determined.

#### Vomer

The vomer forms a single midline element. Its mediolateral width tapers anteriorly. Although the vomer is generally well-preserved, it is broken at various points along its length, and on the midline posteriorly (Figs 4D–E, 5, 6C). It extends anteriorly to the level of the fourth–fifth premaxillary alveoli, and posteriorly to the level of the fourteenth maxillary alveolus, where it contacts the anterior extension of the pterygoids in a mediolaterally broad, deeply interdigitating suture (Fig. 5), as in pliosaurids and most rhomaleosaurids (e.g., [13], [22], [67]–[69]). The vomer-palatine suture curves posteromedially from the posteromedial margin of the internal naris, and is also deeply interdigitating (Figs 5, 11). However, the vomer-maxilla and vomer-premaxilla contacts, which form a continuous line extending anteriorly from the anterolateral margin of the external naris, are only weakly sinuous (posteriorly) and form a loose butt joint (anteriorly) (Figs 4D–E, 5, 6C).

#### Palatine

The palatines are paired elements that form the lateral portions of the palate medial to the maxillae and lateral to the pterygoids (Figs 5, 11). The left palatine is more complete, although its posterior portion has been broken and shifted posteriorly (Fig. 11). The posterior portion of the right palatine has been broken and rotated dorsolaterally. The palatine extends from the internal naris anteriorly, to at least orbital midlength posteriorly, where it is broken. The palatine-pterygoid suture is sinuous anteriorly and interdigitatng posteriorly. It extends posterolaterally from the intersection of the palatine, vomer and pterygoid, approximately parallel to the lateral surface of the skull (Fig. 5). A narrow, elongate notch between the posterior part of the left palatine and the left maxilla represents the anterior end of a suborbital fenestra (Fig. 5). Both palatines are pierced by a number of large foramina.

#### Pterygoid

The pterygoids form most of the posterior palate, and although they are only partially preserved (Fig. 5), it is clear that they followed the typical plesiosaurian pattern in possessing anterior, lateral, and posterior rami. Most of the anterior ramus is preserved on the left side, but is broken at approximately orbital midlength. The right anterior ramus is more fragmentary. The lateral ramus is partially preserved on the right, although its posterior edge, which would have formed the anterior margin of the subtemporal fossa, is broken. A small notch indents its anterior edge, and may have formed a small palatal fenestra between the pterygoid and palatine (Fig. 5). The posterior rami, which would have underplated the basicranium ventrally, are largely broken, although an anterior portion is preserved on the right (Figs 5, 12). Because of breakage, it is impossible to determine whether an anterior interpterygoid vacuity was present. However, an anteroposteriorly long midline separation of the pterygoids anterior to the posterior interpterygoid vacuity exposes the cultriform process of the parasphenoid (Figs 5, 12C) on the ventral surface of the palate, as occurs in most pliosaurids (e.g., [16], [24]–[25], [67]), but not in brachaucheninines, in which the pterygoids meet on the midline immediately anterior to the posterior interpterygoid vacuity, concealing the cultriform process in ventral view (e.g., [79], [26]:character 86).

Only a small anterior portion of the rim of the posterior interpterygoid vacuity of DORCM G.13,675 is preserved (Fig. 12C). However, it is possible to constrain its morphology. The posterior interpterygoid vacuity clearly extended anteriorly of the broken posterior edge of the lateral ramus ( = the anterior edge of the subtemporal fossa), as in some other thalassophonean pliosaurids [16], [54], [79], but not *Peloneustes* [24]. This also occurs in leptocleidids [68], [70], [74], [80] ([69]:character 43).

Although the ventrolateral flange of the pterygoid is broken in DORCM G.13,675, its course can be seen in a strip of broken bone that crosses the ventral surface of the anterior part of the right posterior ramus (Fig. 5). Assuming that the ventrolateral flange met its counterpart on the midline and formed the posterior edge of the posterior interpterygoid vacuity as in other thalassophonean pliosaurids (e.g., [16], [24]), then the dimensions of the vacuity can be estimated as approximately 325 mm long by 145 mm wide. Bone fragments representing possible broken sections of the posterior ramus of the pterygoid are preserved in the matrix dorsal to the basioccipital, lateral to the location of the posterior interpterygoid vacuity, indicating that this region had disintegrated prior to burial.

#### Exoccipital-opisthotic

The left exoccipital-opisthotic is still articulated with the supraoccipital (Fig. 13), but disarticulated from the rest of the braincase and fixed within the left temporal fossa by attached matrix (Figs 2, 5, 13). The right exoccipital-opisthotic has been freed from matrix (Fig. 14). The ventral articular surface for the basioccipital comprises a small anterior portion contributed by the opisthotic, and a large posterior portion contributed by the exoccipital. These contributions are divided by a conspicuous, mediolaterally oriented fissure, indicating only partial fusion of the exoccipital and opisthotic. The body of the exoccipital-opisthotic is dorsoventrally low and anteroposteriorly broad. The posterior ampullary recess is evident in the well-preserved anteromedial surface of the left exoccipital-opisthotic (Fig. 13) and extends onto the articulated portion of the supraoccipital. Semi-circular canals cannot be identified with confidence, although a shelf of broken bone extending laterally from the ampullar recess may indicate the course of the horizontal semi-circular canal. The right exoccipital-opisthotic is less well-preserved, the anterior section having broken away through the line of the metotic or jugular canal (Fig. 14; this canal penetrates the body of the element along the plane of fusion between the exoccipital and opisthotic in plesiosaurians). A circular foramen pierces the medial surface of the exoccipital-opisthotic body (Fig. 14) and is identified as a foramen for the hypoglossal nerve (XII). A corresponding foramen can be seen piercing the wall of the metotic canal, indicating that the hypoglossal nerve exited the exoccipital-opisthotic via the metotic canal. The right exoccipital-opisthotic is broken anterior to the hypoglossal foramen, but an additional smaller foramen can be seen piercing the medial surface of the more complete left exoccipital-opisthotic. Thus the exoccipital-opisthotic body is penetrated by two hypoglossal foramina and the metotic foramen medially, whereas laterally there is a single common foramen, as in pliosaurids and rhomaleosaurids [13], [16], [24], [81]. This is unlike the situation in most plesiosauroids, in which multiple foramina exit laterally (e.g., [73], [82]–[85]). The supraoccipital facet can be seen in the right exoccipital-opisthotic of DORCM G.13,675, with a clear suture separating a small, triangular exoccipital contribution from a larger anterior opisthotic contribution (Fig. 14). The paroccipital process is elongate, with a dorsoventrally broad, spatulate distal half for articulation with the suspensorium (Fig. 14). A rugose ridge extends medially on the posterior surface of the exoccipital between the proximal end of the paroccipital process and the edge of the foramen magnum. This exoccipital flange has previously been called the ‘facet like a zygapophysis’ [16] or ‘atlas-axis articulating facet’ [81].

#### Supraoccipital

The supraoccipital is broken and preserved in two portions disarticulated and embedded in matrix remaining within the left temporal fenestra. The left portion of the supraoccipital is preserved articulated with the left exoccipital–opisthotic (Figs 2, 13D–E). The right is on its own, dorsal to the left portion (Figs 2, 13A–C). The supraoccipital of *P. kevani* is similar to that of *Peloneustes* [16], [24]. Its posterior surface is mediolaterally convex and encloses only a small dorsal portion of the foramen magnum (Fig. 13A–B). The parietal contact is planar and horizontal, and the lateral surfaces slope ventrolaterally (Fig. 13A–B). The opening of the posterior vertical canal of the labyrinth is exposed on the ventrally-facing surface for contact with the right exoccipital-opisthotic (Fig. 13C).

#### Basioccipital

The basioccipital forms the posterior part of the basicranium and bears the occipital condyle (Figs 5, 9, 15). The condyle is large (107 mm wide mediolaterally, 104 mm high dorsoventrally), subcircular, and lacks a well-defined neck to separate it from the basioccipital body (Fig. 15). The dorsolateral surfaces of the condyle are embayed by the exoccipital facets. The condyle’s surface is marked by a number of coarse grooves, but it lacks a notochordal pit. This morphology also occurs in other Late Jurassic pliosaurids (pers. obs. BRSMG Cc332, Cd6172, and [3]), except *Gallardosaurus*, which has a smooth condyle with a well-defined notochordal pit [12], as in Middle Jurassic pliosaurids [16], [24].

In DORCM G.13,675, only the posterior portion of the basicranium is preserved, and is highly abraded. This abrasion has almost completely eroded the pterygoids, revealing the morphology of the dorsally overlying basicranial bones. The abrasion, and associated fracturing, makes it difficult to interpret and to differentiate basicranial elements. A small, diamond-shaped platform is present on the ventral surface of the basioccipital, just anterior to the occipital condyle. This structure, often called the ‘ventral plate’ or ‘ventral process’ probably contacts portions of the parabasisphenoid anteriorly. Well-developed basal tubera project laterally from the body of the basioccipital (Figs 9, 15). These contact the posterolateral processes of the parasphenoid anteriorly and laterally, but no evidence of any more extensive contact is preserved. The basal tubera of most other Late Jurassic pliosaurids also project laterally or only slightly ventrolaterally (BRSMG Cc332, Cd6172; OXFUM J.92451/4), but in brachaucheninines, *Gallardosaurus*, and Middle Jurassic pliosaurids they project further ventrolaterally so their articular surfaces for the pterygoids are located ventral to the occipital condyle [6], [12], [16], [24].

#### Parabasisphenoid

The basisphenoid and parasphenoid are often conjoined in plesiosaurians, and the line of fusion between them can be difficult to discern. The combined parabasisphenoid forms the anterior part of the basicranium, and extends anteriorly into the palate as the cultriform process. (Figs 5, 12) The central portion of the parabasisphenoid, which divides the posterior interpterygoid vacuity, is broken and missing in DORCM G.13,675. Thus, of the parabasisphenoid, only the cultriform process and posterior fragments are preserved. The cultriform process extends far anteriorly on the palate, tapering along its length. Its base is mediolaterally constricted.

The anterior end of the preserved posterior portion of the parabasisphenoid bears a low, abraded midline ridge (Fig. 15). This suggests that the ventral surface of the parabasisphenoid bore a median ridge, as in Middle Jurassic [16], [24] and some Late Jurassic pliosaurids (NHMUK PV OR 39362), but unlike in BRSMG Cc332 [54], *Gallardosaurus* [12], and brachaucheninines [6], [27], [79], in which the ventral surface of the parabasisphenoid lacks a ventral keel.

The cross-section of the parabasisphenoid of DORCM G.13,675 is visible on the broken anterior surface of the basicranium, and shows a dorsal area of highly cancellous bone. This may represent a poorly-ossified basisphenoid contribution to the parabasisphenoid and is seen in some other pliosaurid specimens (e.g., LEICT G418.1956.58.1.4). The portion visible in ventral view consists of denser bone with longitudinal trabeculae, forming the ventral and ventrolateral surfaces of the parabasisphenoid, and likely represents the parasphenoid. This structure continues posteriorly on the ventral surface of the basicranium, contacting the ventral plate of the basioccipital, and producing posterolateral processes that contact the basal tubera.

### The mandible of *Pliosaurus kevani* n. sp

Both mandibles are well-preserved, but most of the symphysis is missing (Figs 16–17). The total missing symphysial length can be estimated as approximately 400 mm, by comparison of the preserved length from the posterior margin of the glenoid (right side  =  1660 mm; left side  =  1625 mm) with the length of the cranium measured from the quadrate to the snout tip (right side  =  2060 mm; left side  =  2030 mm). Compared to its anteroposterior length, the mandible seems dorsoventrally deep (maximum dorsoventral height  =  320 mm at the coronoid eminence), with an estimated length to maximum depth ratio of 6.25.

The posterior portion of the mandibular symphysis is preserved, as determined by anterior inflection of the medial surface, which becomes planar and rugose, and bears the anterior opening of Meckel's canal around midheight. The mandibles are not coossified in this posterior symphysial region. Furthermore, the lateral surfaces of both rami were heavily encrusted with epifauna prior to preparation, while their medial surfaces were almost free of encrustation. This suggests that in both rami rested in the sediment with their lateral surfaces facing upwards. This may indicate that the symphysis was not firmly joined anteriorly, and was thus similar to some brachaucheninines (e.g., [27]) but unlike other Jurassic pliosaurids (e.g., [16]–[17], [23]–[24], [43], [55]). Alternatively however, and perhaps more likely, the symphysis may have been broken prior to burial.

The lateral surface of the mandible is weakly convex dorsoventrally for most of its length. However, posteriorly (where it is formed by the surangular dorsally and angular ventrally), the lateral surface is concave (Figs 16–17).

#### Dentary

The dentary forms much of the body of the mandible anteriorly (Figs 16–17). It is dorsoventrally deep compared to its mediolateral width. The medial surface of the dentary is covered by the coronoid dorsally and splenial ventrally. Together, the coronoid and splenial form the medial wall of Meckel's canal, which excavates the body of the dentary and opens anteriorly in the posterior region of the symphysis (Figs 16–18), between the anterior ends of the splenial and coronoid. The lateral surface of the dentary is weakly convex dorsoventrally, and bears a row of unevenly spaced, anteroposteriorly elongate foramina dorsally. The dentary continues posteriorly as a thin sheet of bone with crenulated edges. This sheet bifurcates posteriorly into two lobes, a posterodorsal lobe that covers the lateral surface of the surangular, and a posteroventral lobe that covers the angular (Figs 16–17). The posterodorsal lobe extends furthest posteriorly, to the level of the coronoid eminence.

As in the maxilla and premaxilla, the dentary alveoli are bounded medially by low, subtriangular paradental plates formed from rugose bone. These plates extend medially to divide the alveoli (Figs 18–19). Replacement alveoli are visible medial to the bases of the paradental plates. The left dentary preserves 27 alveoli, of which five are located within the symphysis. The right dentary, which is slightly more complete, preserves 28 alveoli, of which six are symphysial. Most alveoli open dorsally, as is usual in other plesiosaurians. However, the occlusal surface of the dentary is inclined to face dorsolaterally in the posterior region of the symphysis, so that the fifth–seventh alveoli (as counted from the preserved anterior end of the right dentary) open dorsolaterally (Figs 16–18). This was not observed in any other pliosaurid during the present study and is an autapomorphy of *Pliosaurus kevani*.

#### Splenial

The splenial is an anteroposteriorly elongate sheet of bone that covers the medial surface of the mandible ventrally (Figs 16–17). It extends far anteriorly to form the posterior portion of the mandibular symphysis, as in many pliosaurids and other intermediate- and long-snouted plesiosaurians (e.g., [65], [67]–[69]). It tapers abruptly posteriorly, becoming dorsoventrally narrow and terminating just posterior to the level of the coronoid eminence. Here, the splenial is supported ventrally by a medial buttress of the angular that forms the ventral floor of Meckel's canal. A small, anteroposteriorly elongate foramen penetrates the splenial-angular contact, entering Meckel's canal (Figs 16–17, 20).

#### Coronoid

The coronoid is an anteroposteriorly elongate sheet of bone that covers the medial surface of the mandible dorsally (Figs 16–17). It extends anteriorly to form the posterior portion of the mandibular symphysis, as in many pliosaurids and other longirostrine plesiosaurians (e.g., [22]–[24], [45]–[47], [65], [68], [86]). It extends posterior to the level of the coronoid eminence, where it overlaps the lateral surface of the surangular and terminates in a deeply crenulated, dorsoventrally trending edge (Figs 16–17, 20).

#### Surangular

The surangular forms the dorsal part of the body of the mandible posteriorly (Figs 16–17, 20). Its lateral and dorsal surfaces are covered anteriorly by the dentary, and its medial surface is covered anteriorly by the coronoid. Thus, the anterior extent of the surangular within the mandible cannot be determined. Posteriorly, the surangular contacts the articular, which forms the mandibular glenoid. The dorsal portion of the surangular anterior to the glenoid is transversely expanded and bears a broad, mediolaterally convex, dorsolaterally facing surface that is excavated laterally by an anteroposteriorly elongate fossa with a sharp lateral rim (Fig. 20). The presence of a mediolaterally broad dorsal surface of the surangular is shared with other thalassophonean pliosaurids ([68]:character 87). However, whereas in Middle Jurassic taxa and the Late Jurassic holotype of *P. carpenteri* (BRSMG Cd6172) the resulting broad surface faces dorsally [23]–[24], in most Late Jurassic specimens, including DORCM G.13,675 and the mandible of the holotype of *Pliosaurus brachydeirus* (OXFUM J.9245) it is inclined to face dorsolaterally (BRSMG Cc332; CAMSM J.29561; NHMUK PV OR 39362; OXFUM J.10454). Furthermore, in Late Jurassic specimens except for CAMSM J.29561, the lateral fossa and rim are present, as in DORCM G.13,675.

The surangular of DORCM G.13,675 makes an anteroposteriorly oriented contact with the angular ventrally (Figs 16–17, 20). The plane of this contact is subhorizontal, so it is expressed at the same dorsoventral level both medially and laterally. The surangular–angular contact is penetrated by two oval foramina (Figs 16–17, 20).

#### Angular

The angular forms the ventral part of the body of the mandible posteriorly. It extends anteriorly within the mandible, where it is covered medially by the splenial and laterally by the dentary. However, it remains exposed on the ventral surface of the mandible as a long anterior process dividing the splenial and dentary until just posterior to the mandibular symphysis (Fig. 18). The angular extends posteriorly ventral and lateral to the glenoid and forms part of the retroarticular process (on which its suture with the articular is not visible, and was possibly closed) (Figs 16–17, 20). The left retroarticular process is well-preserved. It is posterodorsally inclined and anteroposteriorly short, subequal to the length of the glenoid. It has a suboval cross section and is expanded slightly towards its posterior end, which is abraded and measures 95 mm dorsoventrally and 75 mm mediolaterally.

Knutsen [34] identified four morphotypes of the retroarticular process in Late Jurassic pliosaurids. The main criterion for recognising these is the shape of the outline in lateral view. The retroarticular process of DORCM G.13,675 seen in lateral view has a ventral surface that curves smoothly posterodorsally, and a dorsal surface that is approximately horizontal (Fig. 20B). The process is mediolaterally much narrower than the glenoid, as in all the morphotypes except for ‘morphotype II’ [34]. However, the morphology does not correspond exactly to any of the morphotypes, and we note that differences in preservation (i.e. deformation and completeness) and ontogenetic stage (the process seems to grow posteriorly through ontogeny) complicate the situation further.

#### Prearticular

The prearticular is a dorsoventrally narrow, anteroposteriorly elongate, band-like bone (Figs 16–17, 20). It originates just anterior to the glenoid, contacting the anteromedial surface of the articular. The prearticular is supported ventrally by the medial buttress of the angular, forming the ventromedial wall of Meckel's canal, which is only partly enclosed in this region (it is open dorsolaterally).

#### Neomorphic mandibular element

A broken, anteroposteriorly oriented band of bone is present on the medial surface of the mandible level with the posterior part of the tooth row (Figs 16C–D, 17C–D, 19B). Examination of cross-sections demonstrates that this is not a portion of the prearticular, and it is tentatively identified as a neomorphic ossification, possibly an analogue of the additional coronoid bone present in some early amniotes. Alternatively, this ossification could be directly homologous with an additional coronoid, in which case an additional coronoid must have been present in more basal pliosaurids and earlier plesiosaurians, but thus far have been overlooked. Whatever its identity, this ossification in DORCM G.13,675 encloses a smooth, rounded rim posteriorly. This is especially well preserved on the right side, and is suggestive of a foramen through which Meckel's canal might have made the transition to being fully enclosed anteriorly to being only partly enclosed posteriorly. A similar opening is present in *Peloneustes* (LEICT G418.1956.33; collected by Phillips in 1923 from the Peterborough Member of King's Dyke brick pit, near Peterborough, United Kingdom). However, in *Peloneustes* it seems to be formed by the coronoid, and evidence for a separate neomorphic ossification is absent.

#### Articular

The articular forms the mandibular glenoid and perhaps a portion of the retroarticular process. However, this is difficult to determine because the angular-articular suture is only visible medially, immediately ventral to the glenoid. The glenoid faces dorsomedially, and consists of an anteroposteriorly long lateral cotyle, and shorter medial cotyle, that are confluent, forming a single, mediolaterally broad depression (Figs 16–17, 20).

### The dentition of *Pliosaurus kevani* n. sp

Several teeth are preserved attached to the skull or deep within the alveoli. Four have been prepared free. The two best preserved are shown in Figure 21, representing a large tooth from the mesial half of the snout (Fig. 21A–E) and a small, distal ‘ratchet’ tooth (Fig 21F–H). All teeth are conical, curved, have approximately circular cross sections with a slightly flattened labial surface ( =  ‘subtrihedral’). Comparison with other pliosaurids suggests the teeth curved lingually. The teeth of DORCM G.13,675 bear numerous coarse, apicobasally oriented ridges. Because the tooth apices are broken, it is impossible to say how far these ridges continued. In all the teeth, the labial surface (which is apicobasally convex) is slightly flattened. This flattening is more pronounced in the mesial teeth (Fig. 21A–E), in which the enamel of the flat surface bears only a few enamel ridges basally. In small, distal teeth, this surface bears a reduced number of relatively prominent enamel ridges (Fig. 21 F–H).

A flattened labial surface has been observed in Late Jurassic pliosaurid teeth by many authors (e.g., [3], [17], [34]–[35], [51], [54]–[55]). This flattening is absent in pliosaurids of other intervals, such as the Early–Middle Jurassic (e.g., [13], [16]–[17], [22]–[24]) and Cretaceous (e.g., [5]–[6], [79]), and is an autapomorphy of *Pliosaurus* (e.g., herein and in [34]). However, in previously reported specimens the flat surface has been broadened, so that is it the broadest surface of the tooth. This results in a triangular cross section of the crown, which is commonly described as ‘trihedral’ [30]. In *Pliosaurus kevani* this flat surface is not broadened (Fig. 21E). We describe this condition as ‘subtrihedral’, and suggest it may be an autapomorphy of *P. kevani*. However, this suggestion is tentative because it may also be present in *Gallardosaurus* based on unconfirmed observations of photographs of the poorly preserved dentition (M.E., unpublished data). The subtrihedral morphology is also present in CAMSM J.35990 [56]–[57], which is referred to *Pliosaurus* cf. *kevani* here (see *Systematic Palaeontology*).

Tooth counts, especially counts of teeth within the mandibular symphysis, are important in pliosaurid taxonomy (e.g., [9], [17], [23]–[24], [34], [43]). Because most of the symphysis of DORCM G.13,675 is missing, we employ three lines of reasoning to estimate the dentary tooth count: (1) comparisons of the preserved portion with other Kimmeridge Clay Formation pliosaurid specimens, which yields an estimate of 5–9 missing alveoli; (2) the length of the missing portion compared to the estimate size of the alveoli, which yields an estimate of 7–9 missing alveoli; and (3) the pattern of interlock between mandibular and cranial teeth, yielding an estimate of 8–9 missing alveoli (Figs 3, 22). Each of these estimation methods has weaknesses. For example, DORCM G.13,675 may have differed anatomically from other Kimmeridge Clay Formation pliosaurids, rendering direct comparisons (method 1) invalid. It is also possible that the estimated 400 mm of missing symphysis length in DORCM G.13,675 is incorrect if, for example, the length from the quadrate to the snout tip has been increased by crushing of the posterior part of the cranium. However, all three methods converge on an estimate of 8–9 missing alveoli, which we consider to be reliable. This leads us to suggest that DORCM G.13,675 possessed 36–37 dentary alveoli, of which 14–15 were symphysial.

#### 1. Comparison with other Kimmeridge Clay Formation pliosaurids

The largest preserved dentary alveoli of DORCM G.13,675 are located just distal to the symphysis (alveoli 8–10 from the preserved anterior end of the right dentary). From here, the alveoli diminish in size both distally and mesially (Fig. 18), so all completely-preserved ‘symphysial’ alveoli have relatively small diameters. However, the anteriormost alveolus of the right dentary, which is only partly preserved, is larger than its immediately mesial alveoli. In two Kimmeridge Clay Formation pliosaurid specimens with well-preserved symphyses, this reversal of the trend of diminishing size occurs at the sixth alveolus (BRSMG Cd6172 [55]; OXFUM J.10545 [17]). Thus, at least five additional symphysial alveoli should have been present in DORCM G.13,675. However, other, less complete, specimens suggest a greater count is also possible. For example five alveoli are preserved mesial to the small, distal symphysial alveoli in NHMUK PV OR 39362, but at least one or two more must have been present in the anterior broken region, suggesting a total of six or seven. Furthermore, the mandible of the holotype of *Pliosaurus brachydeirus* (OXFUM J.9245) preserves seven alveoli mesial to the small distal symphysial ones, and at least one more was originally present, suggesting at least eight (and possibly nine) alveoli could be missing from the right dentary of DORCM G.13,675.

#### 2. Estimate based on the length of the missing portion of the symphysis

Even considering that the missing symphysial alveoli should include the largest in the mandible (e.g., [16]–[17]), if only five were present, as in BRSMG Cd6172 [55] or OXFUM J.10545 [17], then this does not account fully for the estimated missing dentary length of 400 mm: each would measure 80 mm mesiodistally on average, which is very large compared to 50 mm for each of the two largest symphysial alveoli of BRSMG Cd6172 which has a similar skull length (∼1.8 metres [55]) to DORCM G.13,675 (∼2.0 metres). Instead, these measurements of BRSMG Cd6172 suggest that 7–9 alveoli were missing in DORCM G.13,675 (Fig. 3).

#### 3. Interlock of dentary and cranial alveoli

This method relies on the inferring the likely interlocking pattern between the upper and lower dentitions from other pliosaurid specimens [16], [54]. A physical reconstruction of one possibility, in which eight additional symphysial alveoli are hypothesized, has been made by S.M.-F. (Fig. 22). In DORCM G.13,675 the small mesial-most pair of premaxillary teeth would have inserted mesially, between the first pair of dentary teeth. The dentary teeth would then have alternately interlocked with the premaxillary teeth, so that the fifth dentary tooth would have nested between the fifth and sixth premaxillary teeth. A large sixth dentary tooth would have nested into the diastema, and a similarly sized tooth would have nested lateral to the small first maxillary tooth. The dentary symphysis would have been expanded to accommodate the enlarged teeth, and the widest part of the symphysis was likely subjacent to the rostral constriction of the preamaxilla/maxilla. Following this logic, because the preserved anterior end of the right dentary is not laterally expanded, the anteriormost preserved dentary teeth must have nested posterior to the rostral constriction. Thus, at least seven more anterior symphysial alveoli are missing.

The rostral constriction of DORCM G.13,675 is relatively long, extending posteriorly to the mesial margin of the third maxillary alveolus, in contrast to the notch-like constriction seen in *Simolestes* [16], but similar to *Liopleurodon*, or other *Pliosaurus* specimens [16], [54]. This indicates that there was likely at least one, and possibly two, more alveoli mesial to the first preserved dentary alveolus to accommodate a symphysial expansion of comparable length. This results in a realistic maximum count of nine missing alveoli.

## Discussion

### Phylogenetic analysis

We analysed an updated version of the phylogenetic data matrix of Benson & Druckenmiller [26]. The matrix was modified by the addition of five operational taxonomic units representing pliosaurid specimens from the Kimmeridge Clay Formation. This resulted in 85 taxa scored for 270 characters. The data file is available at DRYAD (http://datadryad.org/doi:10.5061/dryad.94f1p). The added OTUs were: the holotype of *Pliosaurus kevani* (DORCM G.13,675), the holotype of *Pliosaurus carpenteri* n. sp. (BRSMG Cd6172 [55]), NHMUK PV OR 39362 [15] (the suggested replacement type of *P. macromerus* [34]), CAMSM J.35991 [52] (the suggested replacement type of *P. brachyspondylus* [34]), CAMSM J.35990 ([57] as *P. macromerus*; but referred to *P.* cf. *kevani* herein), and a specimen from the Chalk near Dorking , Surrey, United Kingdom (DOKDM (Dorking & District Museum, Dorking, United Kingdom) G/1–2; [87], [88]:pp. 20–22, plate 4).

DOKDM G/1–2 was first reported by Owen in 1860 ([87], as *Polyptychodon interruptus*) as being from the Lower Chalk at Dorking, and in the collection of Mr G. Cubitt. Owen later described it again as being in Cubitt's collection, but instead this time coming from a ‘railway tunnel through the chalk formations near Frome, Somersetshire’ ([88]:p. 20). The Frome reference is likely to have been in error; George Cubitt became Lord Ashcombe, his estate was at Denbies, near Dorking, and the specimen is curated at DOKDM, in Dorking. Chalk was quarried in and around Dorking from the West Melbury Marly Chalk, Zig Zag Chalk, Holywell Nodular Chalk and New Pit Chalk formations [89]–[90] of Cenomanian–early Turonian age. Owen's [87] use of the term ‘Lower Chalk’ is likely to have been intended in the traditional sense common in the 19^th^ century. This included only the traditional Chalk Marl and Grey Chalk and so only represents Cenomanian strata [90].

Two characters in the analysis were modified from their usage in Benson & Druckenmiller [26] by the addition of a state (‘2’):

Character 63. Notochordal pit on occipital condyle: absent (0); present (1); occipital condyle scored by multiple pits and deep grooves (2).

Character 139. Cross-sectional shape of teeth in anterior half of tooth row: round or sub-rounded (0); sub-triangular (1;  =  trihedral); intermediate between states 0 and 1, with a flattened labial surface, but this surface not substantially expanded anteroposteriorly (2;  =  subtrihedral).

Tree searches were performed in PAUP* 4.0b10 for Macintosh [91]. Initial exploration for shortest-length tree islands was conducted using four independent randomisations of the Parsimony Ratchet [92] implemented by PAUPRat [93]. The resulting subset of most parsimonious trees (MPTs) was then used as the starting point for TBR (tree bisection and reconnection) branch swapping. ‘Wildcard’ taxa were identified by inspection of the Adams consensus and pruned from the set of most parsimonious cladograms, which were then used to construct a strict reduced consensus [94].

**Figure 23:**
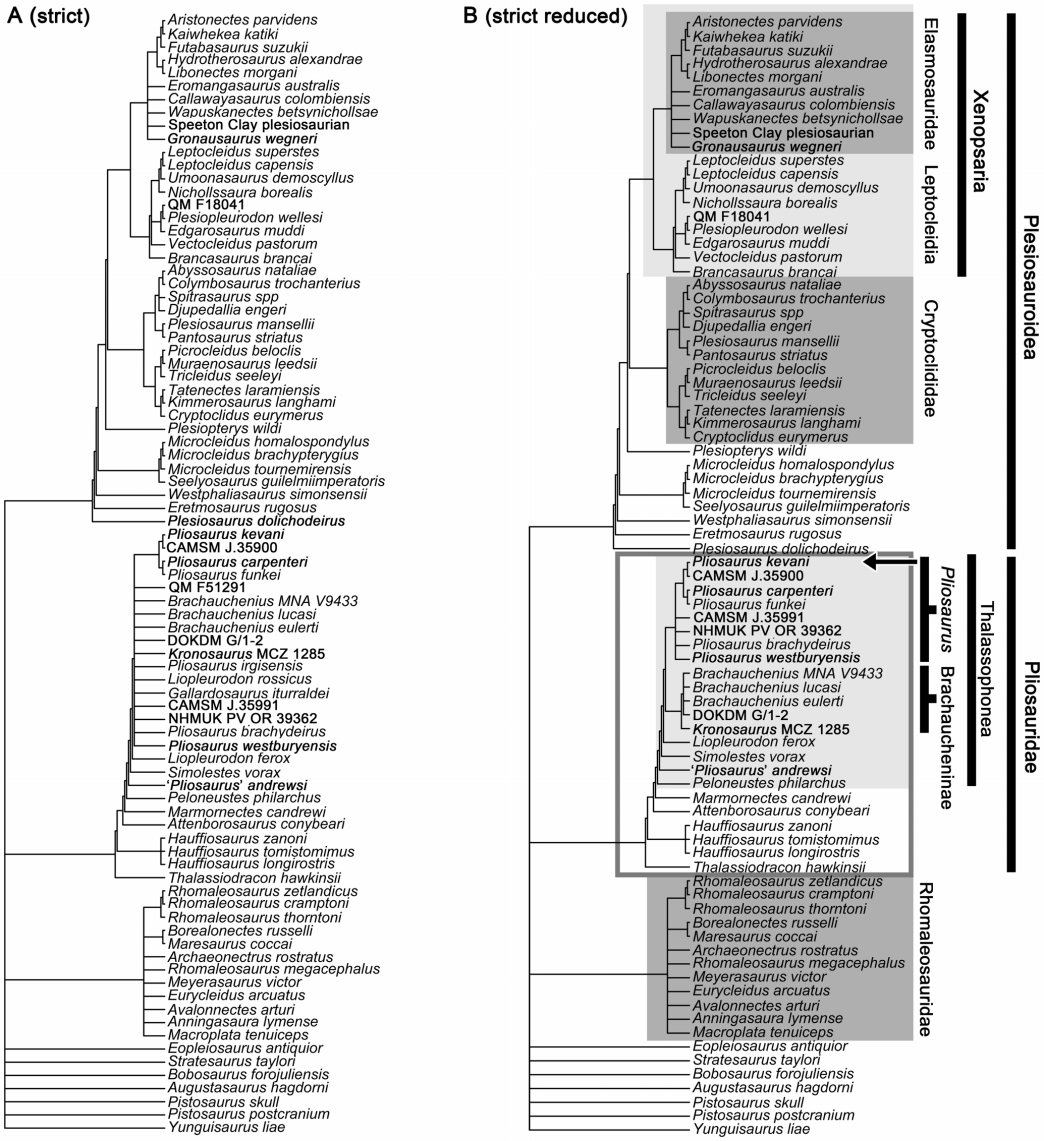
Phylogenetic position of *Pliosaurus kevani* n. sp. DORCM G.13,675. Strict (A) and strict reduced (B) consensus topologies of >130000 shortest length trees 1336 steps long resulting from analysis of our matrix of 85 taxa and 270 characters modified from [26]. Shading is used to indicate major clades, Pliosauridae is enclosed by a grey rectangle, and *Pliosaurus kevani* n. sp. is indicated by an arrow.

Our search recovered >130000 shortest-length trees 1336 steps long. The strict consensus of these trees (Fig. 23) differed from that of Benson & Druckenmiller [26] in two respects. Firstly, within Xenopsaria, several early Cretaceous taxa have slightly different or less resolved positions: *Gronausaurus*, the ‘Speeton clay plesiosaurian’, *Wapuskanectes*, *Callawayasaurus* and *Eromangasaurus* form a basal elasmosaurid polytomy and the leptocleidians *Brancasaurus* [83] and *Vectocleidus* [66], previously found as leptocleidids [26], were found as basal leptocleidians. *Brancasaurus* was found outside of the clade comprising Leptocleididae + Polycotylidae. *Vectocleidus* was found in a polytomy with Leptocleididae and Polycotylidae. This change likely arose from minor scoring amendments and instability of relationships among Early Cretaceous plesiosauroids.

Secondly, most members the thalassophonean clades Brachaucheninae and *Pliosaurus* formed an unresolved polytomy. The exception was a clade comprising two sister taxon pairings: CAMSM J.35990 + *Pliosaurus kevani* and *Pliosaurus funkei* + *Pliosaurus carpenteri*. This polytomy results from the uncertain phylogenetic positions of a few ‘wildcard’ taxa, which occupy multiple positions among the set of shortest-length trees. Pruning these taxa from the trees results in a reduction of the number of unique topologies and an increase in resolution of the strict consensus (a ‘strict reduced consensus’ [94]). Thus, deletion of *Pliosaurus irgisensis* results in resolution of a monophyletic Brachaucheninae in the strict reduced consensus (although relationships within Brachaucheninae remained unresolved). Subsequent deletion of QM F51291 results in resolution of *Kronosaurus queenslandicus* (MCZ 1285) as the sister taxon of an unresolved polytomy comprising three species of *Brachauchenius* plus DOKDM G/1–2 (‘*Polyptychodon interruptus*’ according to [87]–[88]), which we suggest should be referred to *Brachauchenius* indet. Finally, deletion of *Pliosaurus rossicus* and *Gallardosaurus*, in addition to *Pliosaurus irgisensis*, results in resolution of a monophyletic *Pliosaurus* (excluding ‘*Pliosaurus*’ *andrewsi*). The strict reduced consensus after deletion of *Pliosaurus irgisensis, Pliosaurus rossicus*, *Gallardosaurus*, and QM F51291 is shown in Figure 23B.

### Body size of *Pliosaurus kevani*, and pliosaurid body size evolution

The skull of *Pliosaurus kevani* is very large, measuring 1995 mm along the dorsal midline of the cranium and with a mandibular ramus length of 2045 mm excluding the retroarticular process. This is the largest substantially complete Kimmeridge Clay Formation pliosaurid skull (Table 1). However, comparably large individuals have been reported on the basis of more fragmentary material (see Table 1; and explained further below).

**Figure 24:**
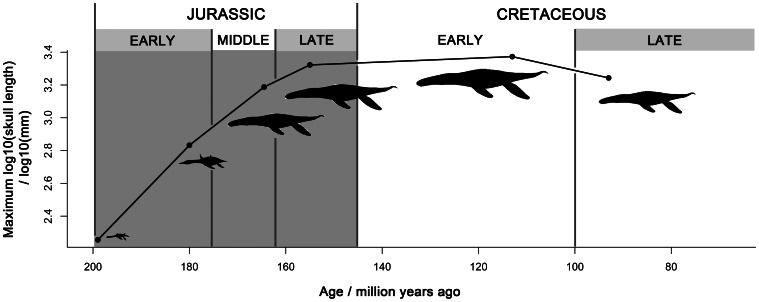
Maximum skull length of Jurassic–Cretaceous pliosaurids. Silhouettes are only approximately indicative of body size and proportions.

Maximum body size of pliosaurids seems to have increased from their first occurrences in the Early Jurassic, until the Early Cretaceous (Fig. 24). Lower Jurassic pliosaurids are relatively small, with maximum skull lengths of 180 mm in the earliest Jurassic (Hettangian) taxon *Thalassiodracon* [13], and 680 mm in the late Early Jurassic (Toarcian) *Hauffiosaurus longirostris* [86]. Plesiosaurian maximum body sizes generally increased during the Early Jurassic, across multiple clades [95]. However, pliosaurid maximum sizes continued to increase after this (Fig. 24). The largest Middle Jurassic pliosaurid, *Liopleurodon ferox* from the Callovian Oxford Clay Formation, has a skull length of 1540 mm [16]. By contrast, most Late Jurassic pliosaurid skulls measure 1700–2100 mm long (Table 1 and below), and some Early Cretaceous pliosaurids were even larger than this ([1], [6], [96] reported skull lengths of at least 2360 mm). However, Late Cretaceous pliosaurids seem to have been smaller than those of the Late Jurassic (maximum skull length of 1750 mm [14]). The Cretaceous plesiosauroid clade Polycotylidae, which independently evolved pliosaurid-like body proportions [18]–[19], never attained sizes similar to the largest pliosaurids. Instead, the largest polycotylids had skulls approximately 1000 mm long [45]–[47] in *Dolichorhynchops bonneri*, *Eopolycotylus*, and *Polycotylus*. This is congruent with the observation that most polycotylids were piscivorous, with long, narrow snouts and gracile, widely-spaced, approximately isodont teeth. Although some polycotylids show slightly more robust, anisodont dentitions, they never evolved especially robust snouts, unlike those of pliosaurids.

At first glance, the Late Jurassic pliosaurid assemblage seems not to consist only of large-bodied species. Some specimens are smaller than 1700–2100 mm long, including the holotype of *Pliosaurus brachydeirus* (OXFUM J.9245), Knutsen's [34] proposed ‘neotype’ of *P. brachyspondylus* (CAMSM J.35991 [52]), and *Gallardosaurus iturraldei* [12] (Table 1). However, these specimens show juvenile features, indicating their small size is the result of incomplete growth, and suggesting that Late Jurassic pliosaurid body size diversity may have been low. These juvenile features include incomplete fusion of the atlas-axis complex (pers. obs. of CAMSM J.35991; and [12]) and possibly incomplete ossification of the proximal convexities of the radius and tibia (CAMSM J.35991; OXFUM J.9245). The cervical and dorsal vertebrae of these specimens preserve disarticulated centra and neural spines, indicating unfused neurocentral sutures, commonly also interpreted as a juvenile feature in reptiles. They also have dorsoventrally short neural spines, with incompletely ossified dorsal ends. This is also indicative of ontogenetic immaturity in sauropterygians [48]. However, the neurocentral sutures remain unfused in very large thalassophonean individuals, including Late Jurassic specimens such as CAMSM J.35990 [57], the holotype of *Pliosaurus carpenteri* (pers. obs. of BRSMG Cd6172) and the large Mexican specimen [2]. Thus, it is possible that vertebral sutural fusion was extremely delayed or never occurred in thalassophoneans. If so, vertebral sutural fusion cannot be used as a reliable indicator of ontogenetic stage in Thalassophonea (*contra* [2]). Alternatively, it is possible that despite their large body sizes, these specimen do represent juveniles, and truly adult specimens have yet to be discovered.

Knutsen et al. [3] found a strong relationship between skull length and the mediolateral width of the occipital condyle in Late Jurassic and Cretaceous pliosaurids. *Pliosaurus funkei* is from the Tithonian (Late Jurassic) of Svalbard. The largest of two individuals of *P. funkei*, PMO (University of Oslo Natural History Museum, Oslo, Norway) 214.136 [3] has an occipital condyle that is 150 mm wide and 105 mm high dorsoventrally. Based on his regression line, Knutsen et al. [3] predicted an original skull length around 2190 mm for PMO 214.136. A slightly lower value around 2040 mm was predicted from the dimensions of cervical centra. It is difficult to compare the size of *P. kevani* with that of *P. funkei* because these taxa are known from different parts of the skeleton. The occipital condyle of *P. kevani* is approximately as high dorsoventrally (104 mm) as that of *P. funkei* (105 mm). However, it is narrower mediolaterally (107 mm compared to 150 mm in *P. funkei* [3]). This suggests either that *P. kevani* is slightly smaller, that *P. funkei* had an unusually broad occipital condyle, or that the occipital condyle of *P. funkei* was taphonomically distorted. Either way, it seems that both individuals were of similar size with skull lengths close to 2 metres.

Other large individuals of Late Jurassic pliosaurids were reported by Tarlo [57] (CAMSM J.35990) and Buchy et al. [2]. The body size of these specimens are difficult to compare to taxa known only from skulls such as *P. kevani*. Although they reinforce the point that many Late Jurassic pliosaurids were large-bodied, they are not suggestive of individuals much larger than *P. kevani* or *P. funkei*. OXFUM J.10454, a highly reconstructed fragmentary pliosaurid skull from Cumnor near Oxford, has a reconstructed total length of 2875 mm [57]. Tarlo [57] suggested that this specimen would have originally been more than 3000 mm long and represented the largest pliosaurid ever recorded. However, the total length cannot be determined and more detailed study is required to establish whether it actually represents an individual substantially larger than other Late Jurassic specimens.

White [6] referred MCZ 1285 from the Early Cretaceous of Australia to *Kronosaurus queenslandicus*. He estimated a skull length of 3720 mm based on comparison with *Liopleurodon* (as ‘*Pliosaurus ferox*’). The total length of this individual, for which most of the vertebral column was known (and now highly restored with plaster) was stated as 12800 mm by Romer & Lewis [1]. Based on the dimensions of its cervical vertebrae, Knutsen et al. [3] estimated the skull length of MCZ 1285 as around 2190 mm. However, this approach employs ‘average’ cervical centrum width, which is taken to mean the average of those preserved, and may be imprecise depending on the pattern of size change along the cervical series. The occipital condyle of MCZ 1285 is 180 mm wide mediolaterally and 125 mm high dorsoventrally (pers. obs. R.B.J.B.), approximately 25% wider and higher than that of *P. funkei*. Using the regression line of Knutsen et al. [3], this suggests a skull length of 2850 mm.

Although the cranial length of MCZ 1285 can only be estimated imprecisely, another Cretaceous specimen, the holotype of *Kronosaurus boyacensis* from the Aptian of Colombia, is more complete [96]. *K. boyacensis* has a midline skull length of 2360 mm and a snout-vent length of 7250 mm. Unfortunately, the tail is missing, preventing comparisons with the estimated total body length of MCZ 1285 provided by Romer & Lewis [1] (12800 mm). However, individual middle–posterior dorsal vertebrae of *K. boyacensis* range from 117–138 mm [96], comparable or only slightly less than 114–145 mm in MCZ 1285 [1]. These dorsal centrum lengths, as well as the skull lengths, of Early Cretaceous pliosaurids, are greater than those of the largest Late Jurassic specimens (dorsal centra range from 100–121 mm in *Pliosaurus* cf. *kevani*; CAMSM J.35990 [57]; most dorsal centra are in the range 97–116 mm in *Pliosaurus funkei* PMO 214.135, only two are greater at 135 and 142 mm [3]). However, the limbs of these Cretaceous specimens seem to be comparable in length, or shorter than, those of the largest Late Jurassic specimens. For example, the humerus is 799 mm long and femur 977 mm long in *K. boyacensis* [96] and the femur is 1060 mm long in MCZ 1285 [1], whereas the humerus is 840 mm long and the femur is 960 mm long in CAMSM J.35990 [57], and the humerus 1000 mm long in *Pliosaurus funkei* [3]. In reconciliation of these observations, Knutsen et al. [3] showed that Early Cretaceous pliosaurids had proportionally short limbs, and that *P. funkei* in particular, has proportionally long limbs compared to other large-bodied pliosaurids. In general, the proportional limb length might decline with increasing body size in pliosauromorphs [18]–[19]. Thus, limb lengths might represent the weakest proxy for body size in pliosaurids.

### Evolution of Late Jurassic pliosaurids

Lack of phylogenetic resolution within *Pliosaurus* and the incomplete nature of many specimens makes it difficult to understand the evolution of Late Jurassic pliosaurids. However, we agree with Knutsen's [34] observation that stratigraphically older specimens, including *Pliosaurus kevani*, have generally longer mandibular symphyses (containing up to 15 alveoli), and younger specimens have fewer (containing as few as six alveoli; e.g., [7], [17], [40]). A reduction also occurs in total premaxillary and mandibular tooth counts (maxillary tooth counts are not confidently known for many specimens), and subtrihedral teeth only occur among the earliest Kimmeridgian (and perhaps in Oxfordian) specimens (Table 1). Some previous authors referred Late Jurassic pliosaurid species with short symphyses to the genus *Liopleurodon* (eg. [42]). Noè et al. [43] explained that there was no good evidence for referral to *Liopleurodon*, but believed that a second, currently unnamed genus was instead present in the Kimmeridge Clay Formation. Our review of the material, phylogenetic results, and observations of low morphological variation among Kimmeridge Clay Formation and other Late Jurassic pliosaurids [26] support a monophyletic group of Kimmeridge Clay Formation pliosaurids, and suggest that there is little basis for defining multiple genera. Therefore, we refer all currently known Kimmeridge Clay Formation pliosaurids to *Pliosaurus*.

*Liopleurodon*, known primarily from the Callovian (Middle Jurassic) Oxford Clay Formation, has a mandibular symphysis comparable in length to some *Pliosaurus* specimens (containing 5–7 alveoli [16]–[17]). However, three observations show that this referral of Late Jurassic specimens to *Liopleurodon* is erroneous: (1) the stratigraphic gap between the Middle Jurassic *Liopleurodon* and the stratigraphically younger species of *Pliosaurus* that show the short symphysis (*Pliosaurus rossicus*, including OXFUM J.10454: Late Kimmeridgian–Tithonian [9], [34]); (2) the absence in *Liopleurodon* of many autapomorphies uniting Late Jurassic pliosaurids within *Pliosaurus* (see *Systematic Palaeontology*); and (3) our phylogenetic results (above) suggest that a short symphysis arose convergently in *Liopleurodon* and *Pliosaurus*.

Thalassophonean pliosaurids were primitively longirostrine with long mandibular symphyses, suggestive of a piscivorous mode of life (e.g., [23]). The independent evolution of short mandibular symphyses in the largest Jurassic genera (i.e. *Liopleurodon* [16] and *Pliosaurus* [34]; and also in *Simolestes* [16]) suggests a functional correlation between macropredatory lifestyles and a short mandibular symphysis. Finite element modelling and beam theory approaches to understanding the mechanical implications of symphysis length in crocodiles indicates that short symphyses perform better under loads used for feeding on large prey [97]. This suggests that symphysis shortening in pliosaurids was an adaptation for macropredation, and is consistent with the appearance of other correlates of macropredation such as trihedral teeth [20] (in *Pliosaurus*) and a strongly anisodont dentition in these taxa.
